# The Force at the Tip - Modelling Tension and Proliferation in Sprouting Angiogenesis

**DOI:** 10.1371/journal.pcbi.1004436

**Published:** 2015-08-06

**Authors:** Patrícia Santos-Oliveira, António Correia, Tiago Rodrigues, Teresa M Ribeiro-Rodrigues, Paulo Matafome, Juan Carlos Rodríguez-Manzaneque, Raquel Seiça, Henrique Girão, Rui D. M. Travasso

**Affiliations:** 1 CFisUC, Department of Physics, University of Coimbra, Coimbra, Portugal; 2 Institute for Biomedical Imaging and Life Sciences (IBILI), Faculty of Medicine, University of Coimbra, Coimbra, Portugal; 3 Department of Complementary Sciences, Coimbra Health School (ESTeSC), Instituto Politécnico de Coimbra, Coimbra, Portugal; 4 Centre for Genomics and Oncological Research: Pfizer/Universidad de Granada/Junta de Andalucía (GENYO), Granada, Spain; K.U.Leuven, BELGIUM

## Abstract

Sprouting angiogenesis, where new blood vessels grow from pre-existing ones, is a complex process where biochemical and mechanical signals regulate endothelial cell proliferation and movement. Therefore, a mathematical description of sprouting angiogenesis has to take into consideration biological signals as well as relevant physical processes, in particular the mechanical interplay between adjacent endothelial cells and the extracellular microenvironment. In this work, we introduce the first phase-field continuous model of sprouting angiogenesis capable of predicting sprout morphology as a function of the elastic properties of the tissues and the traction forces exerted by the cells. The model is very compact, only consisting of three coupled partial differential equations, and has the clear advantage of a reduced number of parameters. This model allows us to describe sprout growth as a function of the cell-cell adhesion forces and the traction force exerted by the sprout tip cell. In the absence of proliferation, we observe that the sprout either achieves a maximum length or, when the traction and adhesion are very large, it breaks. Endothelial cell proliferation alters significantly sprout morphology, and we explore how different types of endothelial cell proliferation regulation are able to determine the shape of the growing sprout. The largest region in parameter space with well formed long and straight sprouts is obtained always when the proliferation is triggered by endothelial cell strain and its rate grows with angiogenic factor concentration. We conclude that in this scenario the tip cell has the role of creating a tension in the cells that follow its lead. On those first stalk cells, this tension produces strain and/or empty spaces, inevitably triggering cell proliferation. The new cells occupy the space behind the tip, the tension decreases, and the process restarts. Our results highlight the ability of mathematical models to suggest relevant hypotheses with respect to the role of forces in sprouting, hence underlining the necessary collaboration between modelling and molecular biology techniques to improve the current state-of-the-art.

## Author Summary

Sprouting angiogenesis—a process by which new blood vessels grow from existing ones—is an ubiquitous phenomenon in health and disease of higher organisms, playing a crucial role in organogenesis, wound healing, inflammation, as well as on the onset and progression of over 50 different diseases such as cancer, rheumatoid arthritis and diabetes. Mathematical models have the ability to suggest relevant hypotheses with respect to the mechanisms of cell movement and rearrangement within growing vessel sprouts. The inclusion of both biochemical and mechanical processes in a mathematical model of sprouting angiogenesis permits to describe sprout extension as a function of the forces exerted by the cells in the tissue. It also allows to question the regulation of biochemical processes by mechanical forces and vice-versa. In this work we present a compact model of sprouting angiogenesis that includes the mechanical characteristics of the vessel and the tissue. We use this model to suggest the mechanism for the regulation of proliferation within sprout formation. We conclude that the tip cell has the role of creating a tension in the cells that follow its lead. On those first cells of the stalk, this tension produces strain and/or empty spaces, inevitably triggering cell proliferation. The new cells occupy the space behind the tip, the tension decreases, and the process restarts. The modelling strategy used, deemed phase-field, permits to describe the evolution of the shape of different domains in complex systems. It is focused on the movement of the interfaces between the domains, and not on an exhaustive description of the transport properties within each domain. For this reason, it requires a reduced number of parameters, and has been used extensively in modelling other biological phenomena such as tumor growth. The coupling of mechanical and biochemical processes in a compact mathematical model of angiogenesis will enable the study of lumen formation and aneurisms in the near future. Also, this framework will allow the study of the action of flow in vessel remodelling, since local forces can readily be coupled with cell movement to obtain the final vessel morphology.

## Introduction

Sprouting angiogenesis—a process by which new blood vessels grow from existing ones—is an ubiquitous phenomenon in health and disease of higher organisms [1]. It plays a crucial role in organogenesis [2], wound healing [3], inflammation [4, 5], as well as on the onset and progression of over 50 different diseases such as cancer, rheumatoid arthritis and diabetes [6, 7]. Currently, many cancer therapies are designed to inhibit the surrounding vasculature depriving the tumor of oxygen and nutrients, but at the same time to facilitate the delivery of chemotherapeutic drugs [8–13]. However, in order to achieve a vasculature that best suits the aim of hindering the tumor development, a detailed knowledge of the regulatory mechanisms of angiogenesis has to be reached. A better understanding of the processes involved in angiogenesis may have a critical impact concerning the strategies to tackle tumor progression as well as in the treatment of several other pathologies where angiogenesis plays an important role.

Angiogenesis is a complex process where a myriad of biological signals, such as the activation of signalling pathways by the binding of growth factors to receptors at the cell membrane, are converted into mechanical forces that originate cell movement. Subsequently, the concerted movement of several endothelial cells lead up to the formation of tubular structures.

Sprouting angiogenesis starts when endothelial cells of existing capillaries acquire the tip cell phenotype by the action of a protein cocktail produced typically by tissue cells in a hypoxic micro-environment [14]. Tip cells lead the growth of new capillary sprouts towards increasing concentrations of relevant growth factors, such as VEGF (vascular endothelial growth factor). Endothelial cells behind the tip cell acquire the stalk cell phenotype, being their proliferation rate regulated by VEGF. The stalk cells that follow the tip cell build the body of the growing sprout [14], and after their spatial rearrangement will form a lumen where blood can flow. When two of these sprouts merge, the blood is able to circulate. The shear forces exerted by the blood flow on the capillary wall trigger signalling pathways in the endothelial cells that lead to further remodelling at the newly formed vasculature, contributing to the shaping of an hierarchical vasculature, with thicker arteries branching into thinner vessels [15–18].

It is rather challenging to study experimentally the articulation between biological and mechanical events in angiogenesis. Recently, some progresses have been reached, for example, the expression of remodelling extracellular proteases and VEGF receptors have been found to be regulated by mechanical stress [18, 19], and there is now a detailed knowledge concerning the mobility of endothelial cells in matrices of different rigidities and subjected to different levels of chemotactic agents [20–22].

Another approach to study the interplay between mechanical and biological processes in angiogenesis is through mathematical modelling. In fact, mathematical modelling is able to combine in a simulation various mechanisms of angiogenesis, thus challenging the current paradigms, and providing new testable hypothesis [23–26]. For research in tumor growth, the use of mathematical modelling is already evident in the community, with complex models able to predict tumor spread [27] and development [28, 29].

The phenomenon of sprouting angiogenesis has been extensively modelled in the last two decades using a large variety of strategies. Both the production of angiogenic proteins and the mechanisms that lead to the formation of complex vascular networks have been simulated in detail. The production of VEGF is intricately dependent on the levels of transcription factors that detect O_2_ levels, such as the Hif-1*α*, and these coupled signalling pathways have been analysed extensively in the modelling literature [30, 31]. The bioavailability of VEGF in the tissue depends on its interaction with the matrix, and this interplay has also been simulated computationally [25, 32–36]. These models enabled a much better understanding of the pathways that regulate the production and signalling of VEGF in angiogenesis.

During these last two decades, research groups have simulated the growth of the new vasculature either via macroscopic continuous models, or using discrete approaches. Continuous models of angiogenesis describe the evolution of average endothelial cell density in a point of the tissue [37–42]. This density field invades the hypoxic tissue while being regulated by the VEGF gradient and/or by the gradient of matrix stiffness (durotaxis). These models allowed the quantitative study of several other biological regulatory mechanisms of angiogenesis such as angiopoietin-1 and angiopoietin-2 levels [42], and were able to monitor the evolution of the pericyte levels in the vessels, thus describing the degree of vascular maturation [41]. However, these models are not able by design to predict the morphology of the growing vascular network. Discrete approaches, on the other hand, aim at modelling the ramified vasculature that results from the angiogenic process [43–46]. An extensive amount of work has been done in these models, where the growing vessels are modelled by reinforced random walks directed towards the gradient of VEGF or other factors in the matrix. Researchers were able to predict vessel flow with these models and to use flow as a further remodelling agent of the vasculature [46]. Discrete models of angiogenesis have been coupled to tumor growth models [47, 48] and used to describe neo-vascularisation in the retina [49]. However they do not model the shape of the growing vessels, and therefore, the interplay with the matrix rigidity is hard to implement. More detailed cell-based approaches, on the other hand, model how individual cells along the vessel interact with each other while they move towards higher VEGF levels [50–53]. These models have much more detail and are able to unravel the mechanisms of cell-cell interactions coupled with signalling pathways in angiogenesis. In spite of cell-based models being able to predict the morphology of the small vasculatures, the large number of parameters and rules often hamper the full exploration of the model’s parameter space. In the last five years, researchers developed hybrid models that combine a continuous description of the vessel sprout with a cell-based approach for tip cell creation and movement [25, 54, 55]. Using techniques such as phase-field modelling, which is able to describe the dynamics of complex boundaries such as capillary walls, these models allowed the study of the shape of vessel sprouts with a smaller number of parameters and rules. However, until now they do not include the ability of matrix rigidity to control tip cell movement. For reviews on the literature of angiogenesis modelling see [17, 56–59].

This effort on angiogenesis simulation has thus provided important insights into this process by unravelling the dynamics of the Notch membrane protein transcription that regulates tip cell selection and the growth and interaction between endothelial cells’ filopodia [52, 60]. Mathematical modeling has helped to identify the role of mechanical traction versus chemotaxis on endothelial cell patterning on a matrix *in vitro* [51, 61]. It has also addressed sprout regression [62] and described the complex movement of cells at the tip of a growing sprout [63, 64]. In this latter case, it has been predicted and observed experimentally the exchange of phenotype between the cells at the front of the sprout, leading to the overtaking of the first cell by the second cell, which becomes the new tip cell. This complex dynamics guarantees the presence of a cell at the front of every growing sprout with the tip cell phenotype that is capable of exerting a pulling force on the matrix, and is characterised by the presence of filopodia capable of sensing the gradient of the angiogenic factors, such as VEGF.

Even after 30 years of research in sprouting angiogenesis there are still many questions regarding the mechanics of vessel sprout elongation and growth that remain elusive. Importantly, the role of the tip cell as the main driver of sprout elongation is still not clear. On one hand the tip cell uses the filopodia to sense the VEGF gradients, attaches itself to the matrix and then exerts a contractile force pulling the cells behind it. On the other hand, the stalk cells proliferate, possibly pushing the tip cell forward. There is still no clear information on the relevance of each of these two opposite mechanisms in the process of vessel sprout growth and elongation. As mentioned, mathematical modelling has already addressed several issues related to tip cell dynamics, and may constitute a suitable approach to elucidate the role of each mechanism in this process.

In this work, we introduce the first phase-field continuous model of sprouting angiogenesis in the literature capable of combining sprout morphology prediction with the elastic properties of the matrix and the forces exerted by the cells. The model we present has the clear advantage of a reduced number of parameters or rules, while being able to run within several partial differential equation solvers.

We use this model to shed light on the regulation by local stresses of endothelial cell proliferation in angiogenesis. We finalize by proposing a mechanism that describes the distribution of forces in a sprout that lead to proper vessel formation.

In the next section we introduce the mathematical model that describes vessel growth coupled with the mechanical characteristics of the tissue. In the Results and Discussion section we start by describing the growth of a single sprout, characterising the conditions for the rupture of this sprout. We then study how vessel proliferation has to be regulated to prevent sprout breakage, and finally we end by drawing the conclusions of this work.

## Models

Phase-field models, originally developed by the physics community in the context of non-equilibrium systems, have achieved great success over the past decades in describing a whole range of materials science phenomena related to nucleation and domain growth [65]. The phase-field permits an elegant and multifaceted numerical description of complex nonlinear problems with moving boundaries. It is a tailorable method, which can be easily adapted to describe quantitatively a vast range of mechanical and dynamical properties of interfaces as a function of bulk properties. Phase-field models are focused on the movement of the boundaries between the domains, and not on an exhaustive description of the transport properties within each domain. For this reason, they require a reduced number of parameters, making them suitable to model morphology and growth of biological systems. So far this type of models has been used in the study of cell shape and movement [66–68], solid tumor growth [28, 29, 69] and angiogenesis [25, 55].

In this work we introduce for the first time a phase-field model of angiogenesis capable of describing vessel sprouting as a function of the mechanical characteristics of the microenvironment. The evolution of the vessel morphology depends also on the concentration of diffusible angiogenic factors. In the present model we consider for simplicity only one diffusible angiogenic factor, which we will deem VEGF, though we can extend this model to include several angiogenic factors [70] and VEGF isoforms that are also able to be captured by the matrix [25, 71]. The gradient of VEGF determines the direction of the tip cell movement. The tip cell is described in the present model by the traction force it is able to exert on the matrix. We consider that the stalk cells do not exert a traction force in the direction of VEGF gradient, due to contact-inhibited chemotaxis [51]. We also include an adhesion force able to be exerted by all cells. The adhesion pulls the cells in the direction of increasing cell density [51], and it is balanced by the corresponding increase in free energy associated to high cell densities.

In detail, an order parameter *ϕ*(**r**) distinguishes between capillaries and the extra cellular matrix (ECM), taking the values *ϕ* = 1 and *ϕ* = −1 respectively. Values of *ϕ* larger than *ϕ* = 1 correspond to areas with high proliferation of endothelial cells which will lead to the widening of the capillary. The position of the capillary wall is given at the level set *ϕ*(**r**) = 0. In the transition between the two phases the order parameter varies continuously within a small distance *ε*. We assume that the dynamics of stalk endothelial cells is the result of the balance between the term describing the dynamics of the vessel and the cell proliferation term, which is regulated by the concentration of angiogenic factor and by the mechanical stresses [25].

In this work we include in the capillary wall dynamics term the effect of the elastic properties of the cells and the ECM. We model these tissues as homogeneous and isotropic, though differing in their rigidity moduli. In this case, the equation for the evolution of *ϕ*(**r**) is given by (see the first 2 sections of S1 Text and [72]):

$$\begin{array}{ccc} \partial_{t}\phi & = & M\;\{\rho_{\phi }\nabla^{2}(-\phi +\phi^{3}-\epsilon^{2}\nabla^{2}\phi )-\mu_{1}\nabla^{2}\;(\sum_{ij}\partial_{ij}w\partial_{ij}w-\frac{1}{d}{\left(\nabla^{2}w\right)}^{2})\\ & + & \frac{\alpha }{L_{0}}\;[\nabla^{2}\chi^{t}+2\mu_{1}\sum_{ij}\partial_{ij}\;(\phi \;\partial_{ij}w-\frac{\delta_{ij}}{d}\phi \nabla^{2}w)]\}+\alpha_{p}\left[V\left(r\right),\phi \left(r\right),w\left(r\right)\right]\;, \end{array}$$ (1)

where *μ*_1_ is the difference between the rigid moduli of the sprout and the ECM, *α* represents the adhesion force between the endothelial cells, and *L*_0_ is an elastic constant related to the average compressibility and rigidity moduli of the endothelial cells and the ECM. In this equation, *M* is the mobility constant that sets the timescale of the problem (see the first section of S1 Text), and *ρ*_*ϕ*_ is a free energy density that determines the balance between the role of the surface tension and of the elasticity in determining sprout morphology and growth. The field *w* depends on the zeroth order displacement field, **u^0^**(**r**), with respect to an unstressed reference configuration: **u^0^**(**r**) = ∇*w*(**r**). The active force exerted by the tip cell, $f^{t}\left(r\right)$, is included through the field *χ* ^*t*^(**r**), such that $\nabla \chi^{t}=-f^{t}$. In the tissue, the displacement field **u^0^**(**r**), and therefore the field *w*(**r**), is a function of the forces exerted by the cells.

**Fig 1:**
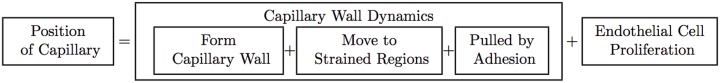
Representation of the terms included in Eq (1). The evolution of the order parameter depends on two processes: the endothelial cell proliferation and the movement of endothelial cells of the capillary on the ECM. The latter results from three main mechanisms that are implemented in our phase-field model. First, the constitution of a capillary wall (cells resist being isolated and leaving the vessel); we model this term as a surface tension. Second, the endothelial cells occupy regions of stiff tissue with high strains; this term is proportional to the difference between the tissue rigidities, but also proportional to the the local strain. Finally the cells can be pulled by the adhesion forces.

In Eq (1) the evolution of the endothelial cells in the tissue depends on the tension along the capillary wall, on the difference in stiffness between the two tissues, on the tissue strain, on the adhesion with neighbouring endothelial cells and on the proliferation (see Fig 1). The derivation of the different terms of the equation is carried out in the first two sections of S1 Text.

We expect that the proliferation of a cell may depend on the average strain along the cell body, and on the concentration of the angiogenic factors at its surface. Therefore, we set the proliferation term *α*_*p*_ as a functional of *ϕ*(**r**), the local strains trough the field *w*(**r**) and the concentration of the angiogenic factor *V*(**r**). In practice, the term *α*_*p*_[*V*(**r**), *ϕ*(**r**), *w*(**r**)] at a specific point **r** corresponds to the average of the proliferation rates in a circular neighbourhood of approximately the size of an endothelial cell around that point. Below we will explore how the proliferation rate depends on the strain and on the VEGF concentration.

Eq (1) is obtained after considering that the mechanical relaxation of the system occurs in a much shorter timescale than the one associated with cell movement. In this quasi-equilibrium approach, the divergence of the stress tensor is related to the external force density, $f_{\mathrm{ext}}$, according to a force balance relation [73] which here simply becomes $L_{0}\nabla \left(\nabla^{2}w\right)=-f_{\mathrm{ext}}$ (see the second section of S1 Text). These external force densities derive from the adhesion forces exerted between the cells (that pull cells together and are controlled by the parameter *α*), and from the traction forces exerted by the tip cells that drive sprout extension, $f^{t}\left(r\right)$. Therefore, *w*(**r**) can be obtained from the following Laplace equation:

$$L_{0}\nabla^{2}w=-\alpha \phi +\chi^{t}\;.$$ (2)

We integrate Eq (1) numerically using a discretised finite differences scheme. We start by fixing the force field created by the tip cell at the front of the growing sprout. At every time step we obtain *w* by solving Eq (2), which is then used to integrate Eq (1) numerically. After a time *t*_*cell*_ ≈ 5 min we find again the location of the tip of the sprout and change the force field to be centred at that site.

The Young’s module of the ECM that is considered in this work is 3.0 KPa, and its Poisson ratio is 0.13 [74]. We refer the reader to the fourth section of S1 Text for the numerical value of the other parameters used in the model. In that section the reader can also find the justification for the values chosen for each parameter.

The VEGF dynamics is also modelled. We consider that it follows a diffusion process with consumption at the vessel [25]:

$$\partial_{t}V=D_{V}\nabla^{2}V-\alpha_{V}V\phi \Theta \left(\phi \right)\;,$$ (3)

where Θ(*ϕ*) is the Heaviside function, *α*_*V*_ is the VEGF consumption rate and *D*_*V*_ is the VEGF diffusion constant in the tissue. The relatively high value of the VEGF diffusion constant in the tissue (see fourth section of S1 Text and [75]) guarantees that the diffusion occurs in a very fast timescale in comparison to cell movement [25].

## Results and Discussion

### Sprout elongation

We start by using this model to study the elongation of a single sprout. Each sprout *in vivo* is lead by a tip cell that exerts a contractile force in the surrounding matrix [20, 61]. The direction of this force is aligned with the direction of the tip endothelial cell polarisation, which can be altered by the angiogenic factors present in the tissue [14]. The traction exerted by the endothelial cells has been measured experimentally in detail [20], and is approximately radial and directed towards the center of the cell body. The contraction of the tip cell is able to produce the observed movement of the sprout in the direction of the angiogenic factor’s gradient.

**Fig 2:**
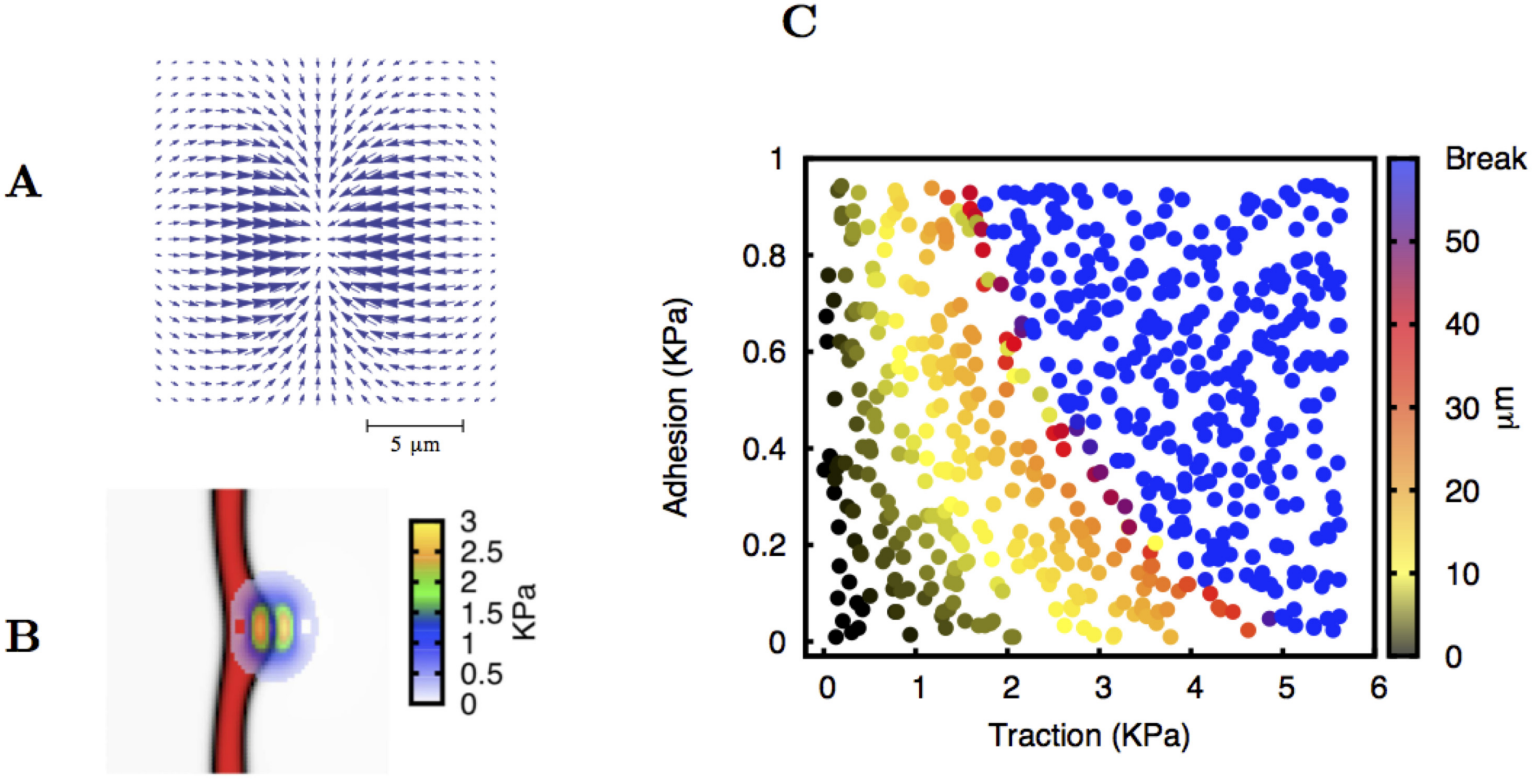
Formation of a single sprout without endothelial cell proliferation. Plate A: The contractile force field that is created by the tip cell is oriented along the direction of the VEGF gradient. Here we consider this force distribution, akin to the traction force field observed by *in vitro* experiments [20]. The typical size of the force region is the same as the size of an endothelial cell. Plate B: The centre of the force field is located at the border of the original vessel. In this figure a sprout is already forming. The local intensity of the traction force exerted by the tip cell is indicated. The maximum traction force in this case is 3.0 KPa. Plate C: Length of the sprout after 14.5 hours plotted as a function of the maximum tip cell traction force and adhesion coefficient. After that period of time we observe that the sprout almost does not increase its size. The values for adhesion and traction tested are chosen randomly within the studied interval. The length of the sprouts increases with the traction force applied. For large values of adhesion and traction force the tip cell pinches off.

In our simulation we start with a vessel directed vertically and located on the left side of the simulated region. We consider a gradient of VEGF that is perpendicular to this vessel. The levels of VEGF are defined relative to the VEGF concentration at the hypoxic cells, on the right boundary of the simulation unit, where we set *V* = 1 as a boundary condition. In this continuous model, the effect of the tip cell is solely modelled by the force it exerts on the tissue. According to what it is observed experimentally, we use a contractile force aligned preferentially with the VEGF gradient (see Fig 2A and the third section of S1 Text). Here, we keep the force profile as simple as possible: centred at the vessel boundary and different from zero within a circular region with 5 *μ*m radius (approximately the radius of an endothelial cell). We initially position the center of the traction force field at a site on the right boundary of the vessel (Fig 2B). Hence, this force mimics the distribution of forces exerted by a tip cell [61].

The movement of endothelial cells to regions where the matrix has larger strains is used in the literature to model tip cell movement on matrices [61]. Similarly, in our model the soft tissue (the vessel) will always move to the regions where the more rigid tissue is being compressed in order to minimise the free energy (see the first section of S1 Text). Therefore, the endothelial cells move to occupy the neighbouring regions where the ECM is strained, since the vessel’s rigidity modulus is lower than the rigidity modulus of the ECM. They will also follow the local gradient of matrix rigidity (durotaxis) [59]. As the vessel is being pulled by the traction force exerted by the tip cell, the back part of the vessel also becomes curved, as it is also pulled forward (Fig 2B). After some time (*t*_*cell*_), we find the new location of the interface between the vessel and the ECM, and reposition the force field in a way it is centred at this new location.

We observe that in most situations, due to the surface tension term, the growing velocity of the sprouts decreases sharply, achieving values below than 2 *μ*m/hr within less than 8 hours. Therefore the vessel effectively stops growing. In Fig 2C we represent (in a color scale) the observed elongation of the sprout after 14.5 hours (effectively the maximum sprout length) for different values of the maximum traction force exerted by the tip cell and the value of the adhesion *α*. To create this figure we simulate 640 different sprouting events with the values of traction and adhesion assigned randomly in the intervals [0, 6.0] KPa and [0:1.0] KPa respectively. For each run we measured the total length of the sprout if it was intact, or recorded if the tip cell broke away from the original vessel. We observe that for a given value of the adhesion, the larger the traction force, the larger is the resulting sprout. However, for very large tractions, the sprout breaks and its tip separates from the vessel and continues the migration.

In the simulation we observe that for a given value of the traction force exerted by the tip cell, an increased value of the adhesion between the cells also leads to a larger vessel. The adhesion allows the cells of the vessel that are behind the tip cell to follow the leading sprout. Also, if the forces that link the tip cell to the cells behind are very low, when the tip contracts it cannot pull the rest of the sprout, and therefore the vessel will not grow. In that situation, the contraction of the tip cannot lead neither to sprout extension nor to vessel rupture. This is exactly what is observed: we obtain smaller vessels for low adhesion and low traction forces. In particular, for a smaller adhesion coefficient, the traction force required to grow a sprout up to a specific length is higher. On the other hand, if the adhesion is very large, when the tip cell contracts, the vessel behind follows readily the tip cell and eventually breaks up (Fig 2C).

Importantly, vessel sprouts that grow indefinitely without breaking (i.e. that are able to maintain their growing velocity) were never observed in our simulations without accounting for endothelial cell proliferation.

**Fig 3:**
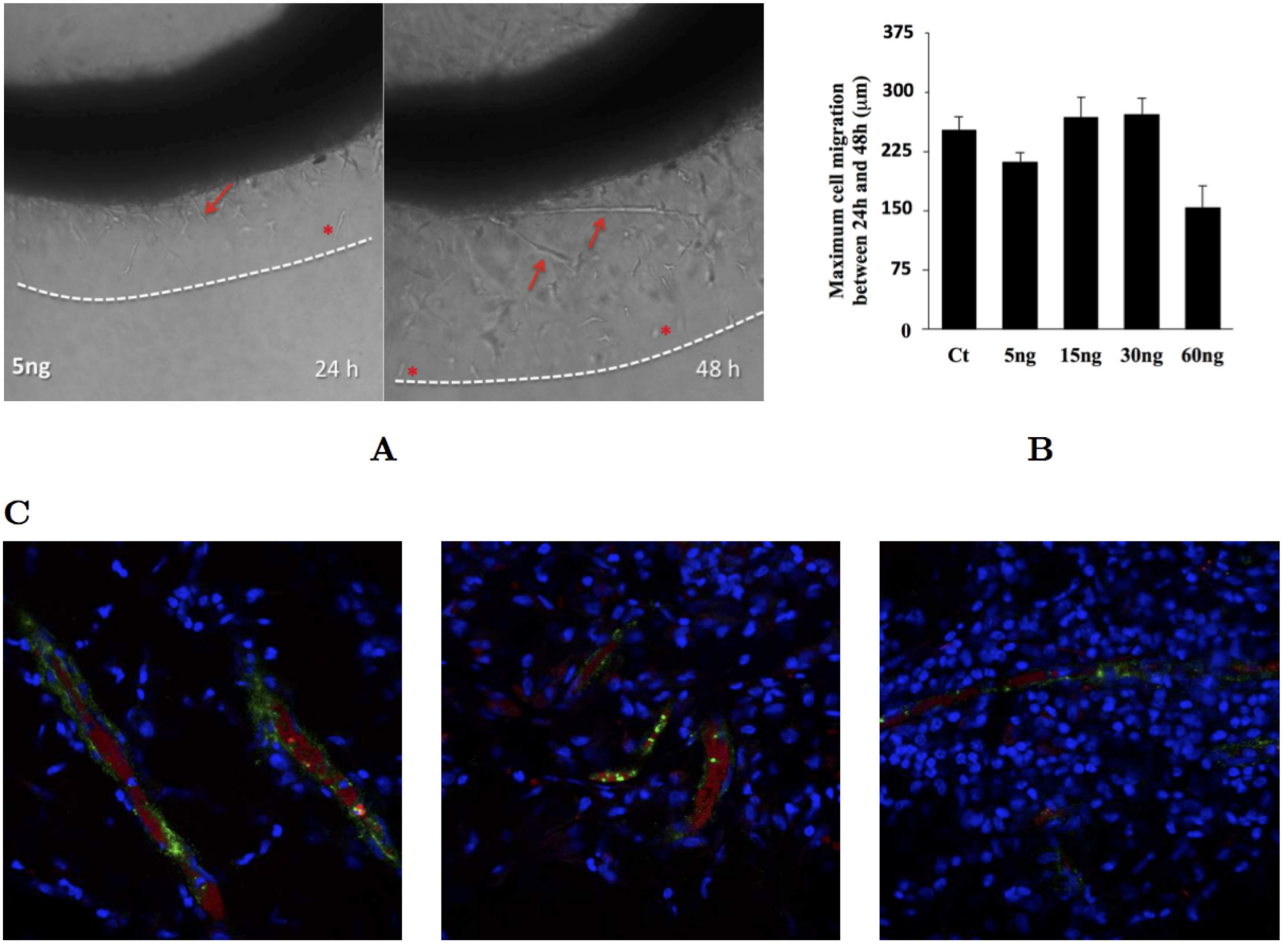
Examples of vessel sprouting *ex vivo* and *in vivo*. Plate A: Aortic ring assay. In this experiment a circular segment of a rat aorta is immersed in a collagen matrix (see sections five and six of the S1 Text for materials and methods related to these experiments). In the following days new sprouts (arrows) form and grow out the ring. Isolated endothelial cells (stars) also leave the vessel and migrate in the matrix. Plate B: Maximum distance travelled by an isolated endothelial cell for different values of the initial VEGF concentration in the medium. We observe that this distance is independent of the concentration of VEGF (except for very high concentrations, where the receptors are all overloaded). We use the velocity of these cells to parameterise the model (see section 4 of S1 Text) Plate C: Matriplug assay. In this system, 0.6 ml of Matrigel are injected in the back of a rat. During the next 7 days, endothelial cells invade the matrigel and blood starts flowing. After these 7 days the Matrigel was excised and observed using a confocal microscope. In these figures the cells nuclei are marked with DAPI (in blue), the perfused vessels are marked with Evans Blue (in red) and the endothelial cells are marked with anti-von Willebrand factor (in green). We observe several functional vessels, but not isolated endothelial cells.

Experimentally, isolated endothelial cell migration is observed in *ex vivo* assays (Fig 3A and 3B). In the conditions of the aortic ring experiment preformed, these cells migrate approximately a distance of 230 *μ*m, independently of the initial concentration of VEGF in the medium (Fig 3B). We can use this fact to fit the time scale of the model, and the value of the parameter *M* that we will use in our simulations (see section 4 in S1 Text). Nevertheless, we do not observe isolated endothelial cells in Matrigel plugs inserted in live mice (Fig 3C). That may occur because the values of the *in vivo* cell adhesion, traction force and the tissue Young’s modulus fall within in a region where the sprouts do not break up, because the cell proliferation is able to prevent tip cell migration (see below) or because the original isolated endothelial cells have died, since they need to be in contact with each other for survival *in vivo*. Below we will explore the role that endothelial cell proliferation has in allowing the growth of long sprouts without breaking up.

### Regulation of proliferation in sprouting angiogenesis

Endothelial cell proliferation is necessary to overcome the short ramification phenotype that we observed in the previous section. It has been reported that the endothelial cells with the stalk phenotype increase their proliferation rate when in the presence of VEGF [14]. On the other hand, it is also known that mechanical stresses can alter significantly the behaviour of endothelial cells, with shear stress being an important trigger factor in vessel remodelling and in endothelial cell proliferation [16–18, 76–78]. Therefore, it is relevant to understand the role of the concentration of angiogenic growth factors (e.g. VEGF) and mechanical stresses in regulating endothelial cell proliferation. With the present model we can test different possible regulatory mechanisms of VEGF and mechanical stresses, and observe the resulting morphology of the vessel sprouts.

Since the presence of individual cells is often seen experimentally (Fig 3A), in the following simulations we start by setting fixed values for the maximum traction and adhesion that lead to the sprout breaking in the absence of proliferation.

It is also observed experimentally that the maximum traction exerted by the tip cell is normally on the order of the magnitude of the matrix’s Young’s modulus (see for example [20, 21]), and so we chose a maximum traction force of 3.0 KPa (approximately equal to the Young’s modulus of the matrix), and *α* = 0.47 KPa (sufficient to have individual cell migration at maximum traction force of 3.0 KPa, according to Fig 2C).

In the remainder of this section we explore several possible mechanisms for regulating endothelial cell proliferation and their consequences regarding vessel formation. We expect that the proliferation is able to prevent isolated tip cell migration. However, endothelial cell proliferation should be located in specific sites for the resulting vessel to grow straight. We consider four different ways for the VEGF concentration and the strain to regulate the proliferation rate, and compare the shape of the resulting sprouts.

**Fig 4:**
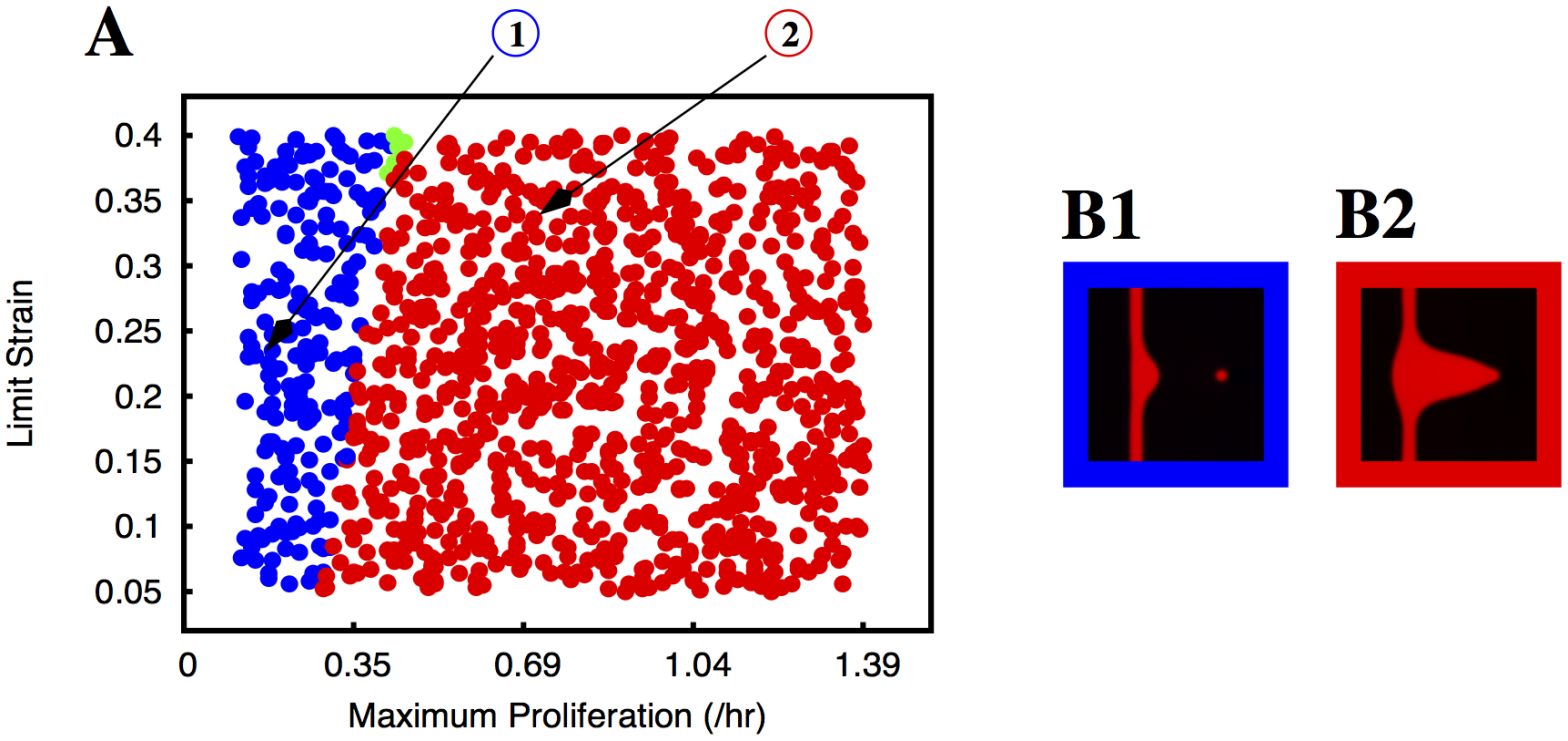
Morphology of the growing sprouts with endothelial cell proliferation dependent on local strain. In plate A we indicate the morphology of the growing sprout (by using different colours) for different values of maximum proliferation (*M*_*P*_) and limit strain (*L*_*S*_). In these simulations the proliferation rate increases linearly with the strain until it reaches the value *M*_*P*_ at the strain *L*_*S*_. The colours indicate the morphology of the observed sprouts for the corresponding parameters: blue dots correspond to situations where the sprout is split and the red dots correspond to deformed vessels (i.e. triangular sprouts). The few green dots correspond to well formed sprouts without appreciable thickening of the parental vessel. We observe that the sprout breaks for low proliferation rates, and it becomes deformed for large proliferation rates; the region of parameters that produce good sprouts is extremely small. In plates B1 and B2 we plot examples of the morphologies observed (the color of the border in these plates follows the same code as in plate A). The parameters used to obtain the morphologies depicted in B1 and B2 are indicated by arrows in plate A.

**Fig 5:**
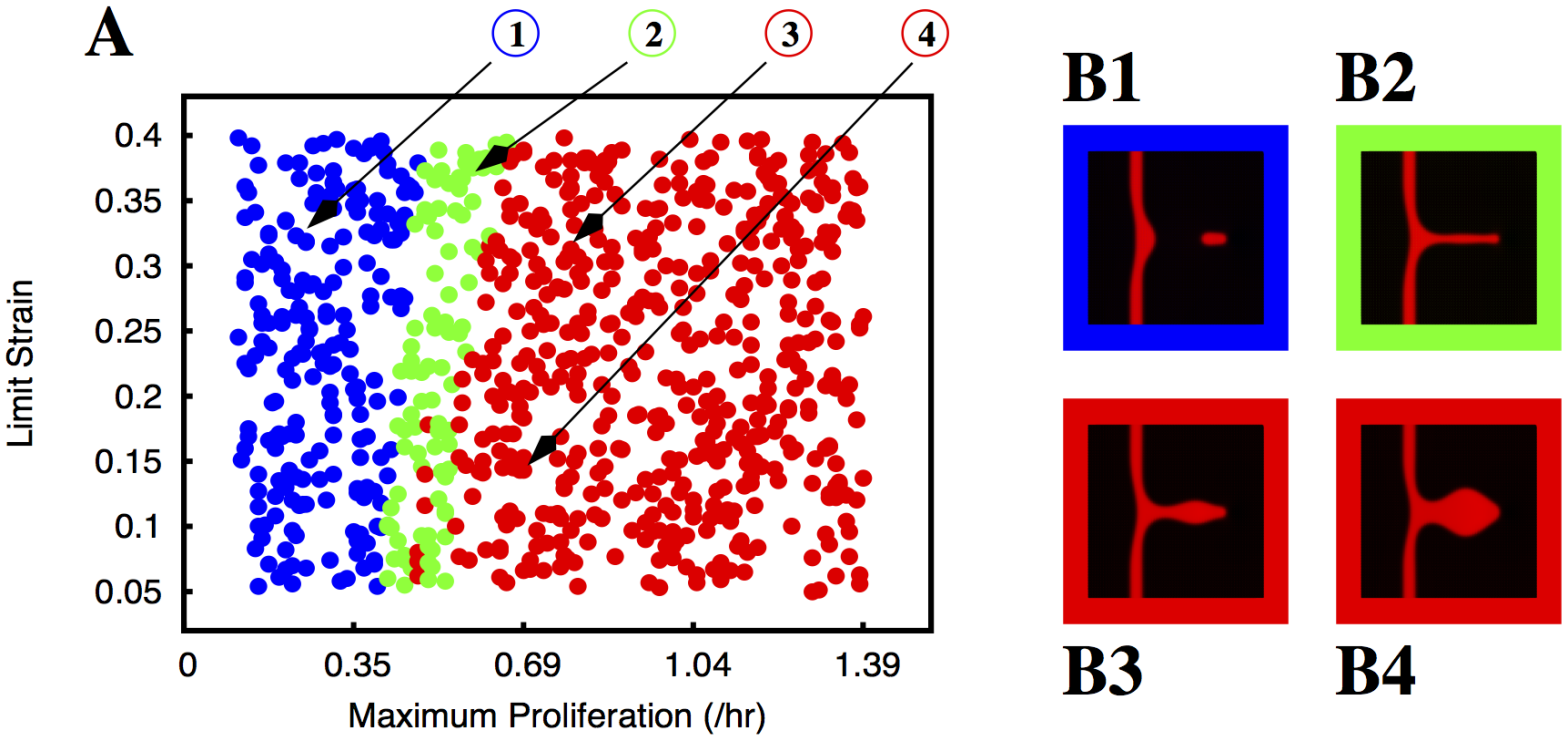
Morphology of the growing sprouts with endothelial cell proliferation dependent on local strain but triggered by VEGF. In plate A we indicate the morphology of the growing sprout (by using different colours) for different values of maximum proliferation (*M*_*P*_) and limit strain (*L*_*S*_). In these simulations the proliferation rate increases linearly with the strain until it reaches its maximum value *M*_*P*_ at the strain *L*_*S*_, but is only different from zero where the concentration of angiogenic factor is larger than a cut-off, i.e. *V* > *V*_*m*_ = 0.05. The colours indicate the morphology of the observed sprouts for the corresponding parameters: blue dots correspond to situations where the sprout breaks, the red dots correspond to deformed vessels (vessels with variable thickness in this case), and the green dots correspond to well formed sprouts without appreciable thickening of the parental vessel. We observe that the sprout breaks for low proliferation rates, and it becomes deformed for large proliferation rates; the region of parameters that produce good sprouts is localised in a narrow vertical band. In plates B1, B2, B3 and B4 we plot examples of the morphologies observed (the color of the border in these plates follows the same code as in plate A). The parameters used to obtain the morphologies depicted in B1, B2, B3 and B4 are indicated by arrows in plate A.

**Fig 6:**
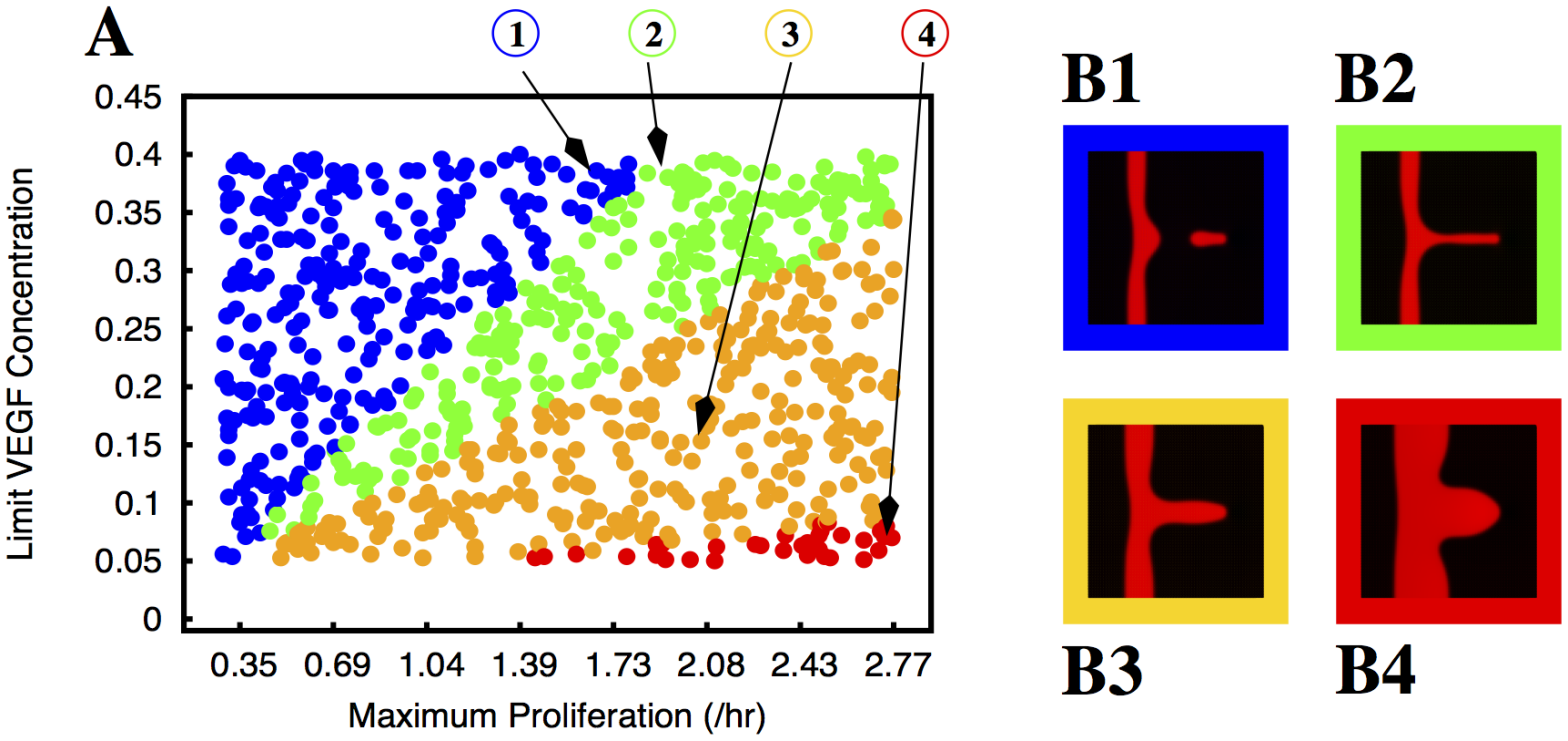
Morphology of the growing sprouts with endothelial cell proliferation dependent VEGF concentration. In plate A we indicate the morphology of the growing sprout (by using different colours) for different values of maximum proliferation (*M*_*P*_) and limit VEGF concentration (*L*_*V*_). In these simulation the proliferation rate increases linearly with the VEGF concentration until it reaches the value *M*_*P*_ at the VEGF concentration *L*_*V*_. The colours indicate the morphology of the observed sprouts for the corresponding parameters: blue dots correspond to situations where the sprout breaks, the green dots correspond to well formed sprouts without appreciable thickening of the parental vessel, the orange dots correspond to well formed sprouts with appreciable thickening of the parental vessel, and the red dots correspond to deformed vessels (triangular sprouts in this case). We observe that the sprout breaks for low proliferation rates, but there is a large region of the parameter space with well formed sprouts. There is however extensive parental vessel thickening. In plates B1, B2, B3 and B4 we plot examples of the morphologies observed (the color of the border in these plates follows the same code as in plate A). The parameters used to obtain the morphologies depicted in B1, B2, B3 and B4 are indicated by arrows in plate A.

**Fig 7:**
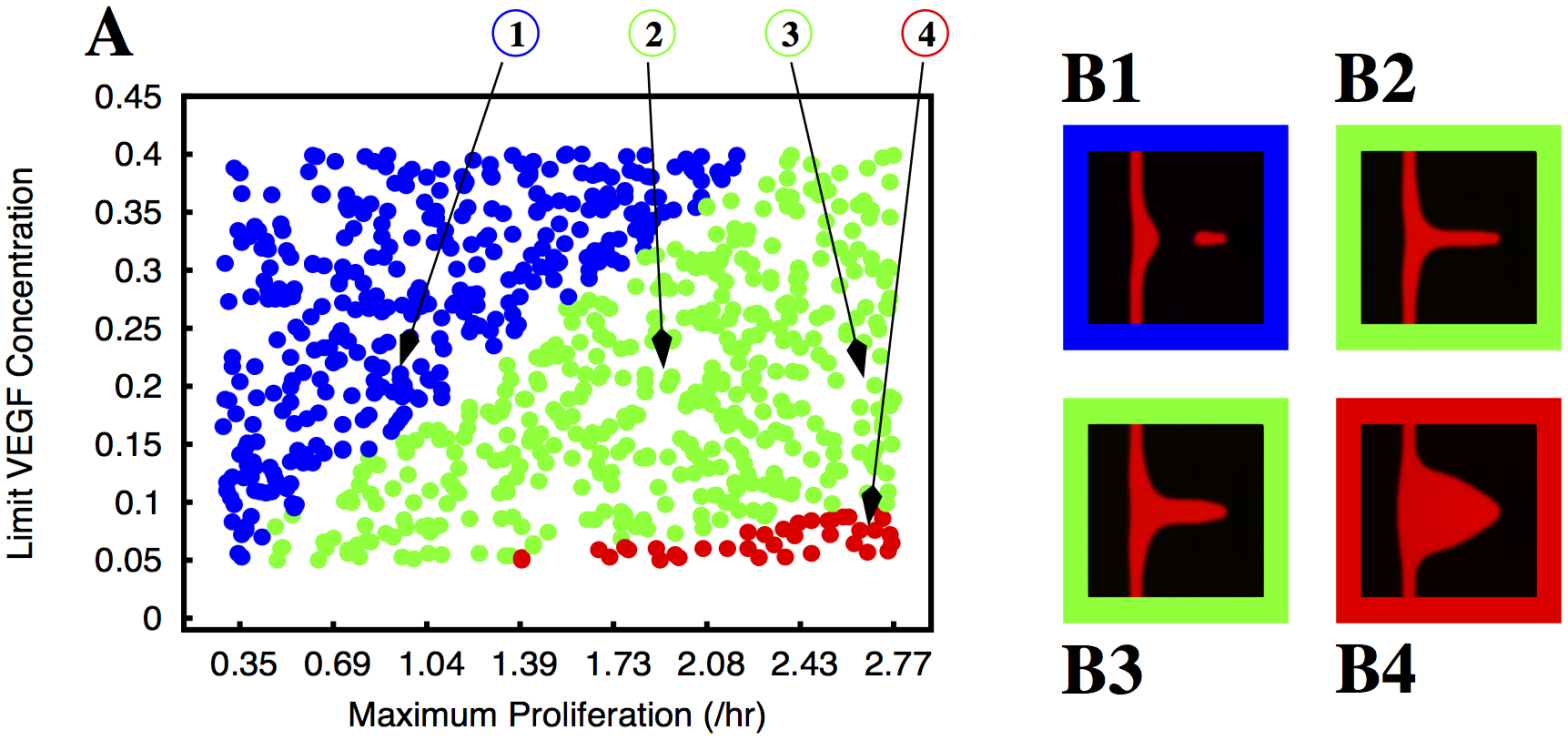
Morphology of the growing sprouts with endothelial cell proliferation dependent on VEGF concentration but triggered by local strain. In plate A we indicate the morphology of the growing sprout (by using different colours) for different values of maximum proliferation (*M*_*P*_) and limit VEGF concentration (*L*_*V*_). In these simulations the proliferation rate increases linearly with the VEGF concentration until it reaches its maximum value *M*_*P*_ at the concentration *L*_*V*_, but is only different from zero where the strain is larger than a cut-off *S*_*m*_ = 0.05. The colours indicate the morphology of the observed sprouts for the corresponding parameters: blue dots correspond to situations where the sprout breaks, the green dots correspond to well formed sprouts without appreciable thickening of the parental vessel, and the red dots correspond to deformed vessels (i.e. triangular sprouts). We observe that the sprout breaks for low proliferation rates, but there is a very large region of the parameter space with well formed sprouts. In plates B1, B2, B3 and B4 we plot examples of the morphologies observed (the color of the border in these plates follows the same code as in plate A). The parameters used to obtain the morphologies depicted in B1, B2, B3 and B4 are indicated by arrows in plate A.

- **Proliferation regulated by local strain.** We start by considering that the proliferation only occurs in the regions of the vessel where the cells have positive strain, i.e. where the cells are being stretched. In here, we consider that the proliferation rate increases linearly with the strain until it achieves the maximum proliferation (*M*_*P*_), at a particular strain value we deem the limit strain, *L*_*S*_. To measure the strain we use the divergence of the zeroth order displacement vector, i.e. ∇ ⋅ **u^0^** = ∇^2^ *w* = −*αϕ*+*χ* ^*t*^. The areas where the cells are stretched have ∇^2^ *w* larger than its equilibrium value in the capillary (corresponding to *ϕ* = 1), i.e. −*αϕ*+*χ* ^*t*^ > −*α*. Hence, the strain that is referred to in Figs 4A and 5A corresponds to *α*(1 − *ϕ*) + *χ* ^*t*^. In the sprout, the region of positive strain is small and localised behind the tip cell, with the first stalk cells being the ones that are typically stretched the most. In Fig 4A we identify the type of observed sprouts for the different values of *M*_*P*_ and *L*_*S*_. Different types of sprouts are identified with different colours. It is clear from the graphic that the resulting morphology does not significantly depend on the value of the limit strain. The site in the vessel with large positive values of strain is small, and the strain varies dramatically in that small region of the vessel. Therefore, considering different values of the limit strain (i.e, the strain at which the maximum proliferation is reached) does not alter significantly the spatial region where the maximum proliferation is attained, leading to a similar sprout morphology. However, the value of *M*_*P*_ influences dramatically the resulting morphology: for low values of *M*_*P*_ the tip cell is still able to break apart from the growing sprout (Fig 4B1), while for values of *M*_*P*_ larger than ≈ 0.35 hr^−1^, the vessel does not break up, but it adopts a triangular shape (Fig 4B2). When the proliferation is controlled solely by the strain, it occurs very far back in the vessel, and therefore the vessel widens significantly at the back.
- **Proliferation regulated by local strain, but triggered by VEGF.** Similarly to the previous type of regulation, in this case we consider that the proliferation is still proportional to the strain (until it reaches the maximum proliferation at the strain value of *L*_*S*_), but we only allow proliferation if the concentration of VEGF is larger than a certain cut-off. Here we set this cutoff to 0.05 (relative to the concentration of VEGF at the right end of the simulating box). In this way, a small concentration of VEGF serves as a trigger for proliferation. Similarly to the previous case, we observe that the type of vessel still does not depend significantly on *L*_*S*_. We also still observe that for low values of *M*_*P*_ there is individual cell migration, while for large maximum proliferations the vessel is malformed (Fig 5A). However, since VEGF serves as a trigger for proliferation, the proliferation occurs preferentially in the sites with higher VEGF concentration, i.e. closer to the front of the vessel. In this way, instead of the malformed vessels adopting a triangular shape, they are not straight, presenting a thicker section at the middle of the vessel than at their root (Fig 5B3 and 5B4). There is however a very short band of values for the parameters where the proliferation is enough to prevent the vessel from breaking up and small enough to not present the bulging at the front. These vessels are considered well formed (Fig 5B2).
- **Proliferation regulated by VEGF concentration.** Another type of vessel proliferation regulation is admitting that it increases linearly with the VEGF concentration until it reaches the maximum proliferation, *M*_*P*_, at a limit VEGF concentration, *L*_*V*_. The higher concentration of VEGF in the sprout occurs at its tip, and therefore in this scenario the new material resultant from the proliferation appears primarily at the front of the vessel, extending it and resulting in sprouts that are straighter than in previous scenarios (Fig 6A). Since the VEGF concentration varies smoothly along the ECM, the value of the limit VEGF concentration has an important role in determining the final morphology. If this value is small, the maximum proliferation is achieved at very low VEGF concentrations and, therefore, large regions of the sprout reach *M*_*P*_. On the other hand, if the *L*_*V*_ is large, the proliferation increases slowly with VEGF until it reaches *M*_*P*_ only at large values of VEGF concentration. In this case, the regions of the sprout with high proliferation rate are solely the ones with high VEGF levels. Accordingly, the sprout morphologies associated with low proliferation are found for low values of *M*_*P*_ and high values of *L*_*V*_. In this region of the parameter space the sprout still breaks (Fig 6B1), leading to individual endothelial cell migration. As the proliferation rate increases, the sprouts stop breaking, and we observe vessels that are straight in the majority of the regimes tried (Fig 6B2 and 6B3). Only at very large proliferations (high *M*_*p*_ and low values of *L*_*V*_) does the sprout becomes deformed and triangular (Fig 6B4). However, when we consider that the endothelial proliferation is proportional to the VEGF concentration, all the cells proliferate, and not only the cells at the sprout. At the parental vessel, the concentration of VEGF is low but different from zero, thus this vessel is also able to become thicker, as it is clearly observed in our simulations for the larger proliferations (see Fig 6A, red and orange points, and Fig 6B3). This thickening of the original vessel is clearly not normally observed during sprouting angiogenesis, where the developing is normally centred at the growing sprout [14, 79]. Moreover, while in sprouting angiogenesis *in vivo* there is no proliferation of the tip cell [14], in the present hypothesis the larger proliferation occurs at the sprout tip. Therefore this hypothesis allocates a proliferation rate that is too high at the tip of the sprout and at the parental vessel.
- **Proliferation regulated by VEGF concentration but triggered by strain.** Finally we consider that there is only proliferation in the areas where the cells are being stretched. In this scenario, the proliferation is linearly proportional to the VEGF concentration until the maximum proliferation *M*_*P*_ is reached (at VEGF concentration *L*_*V*_), but only if the strain is positive and larger than a cut-off, which we set as *S*_*m*_ = 0.05. This small strain, which serves as a trigger for proliferation, is equal to the smallest value of *L*_*S*_ tested in Figs 4A and 5A. For low values of the proliferation (low *M*_*P*_ and high *L*_*V*_) the tip cell still separates from the sprout. However, as the proliferation increases, the region in parameter space with well formed vessels is extremely large (Fig 7A). As in the cases where the proliferation was regulated by the strain, here the parental vessel never becomes thicker (Fig 7B2, 7B3 and 7B4). Even for the largest proliferation rates, where the vessels become triangular for very high *M*_*P*_ and low *L*_*V*_, the parental vessel is thin and the proliferation is confined to the growing sprout (Fig 7B4). Most of the region of the parameters tested either produces vessels that are straight or vessels that break off (at low proliferation rates). Malformed triangular sprouts only occur for very large proliferations.

**Fig 8:**
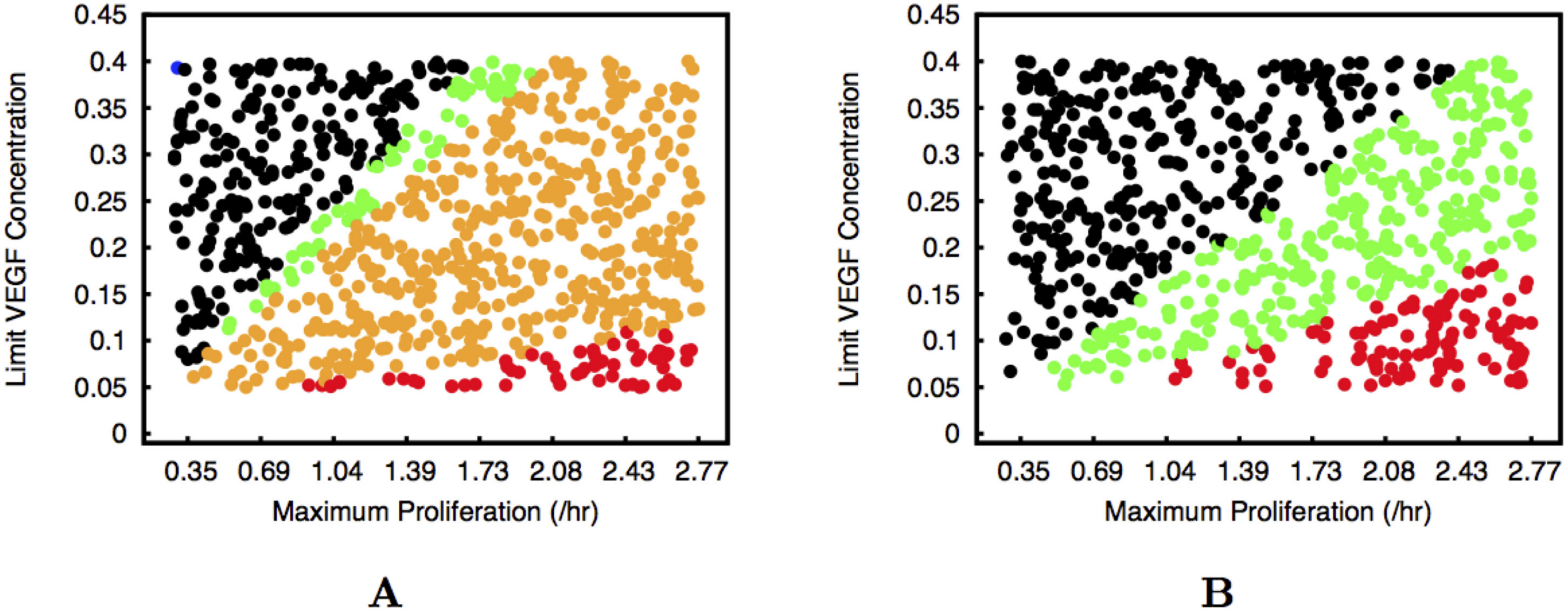
Morphology of the growing sprouts with low adhesion between endothelial cells. The two plates correspond to the cases of proliferation regulated by VEGF concentration (Plate A), and proliferation regulated by VEGF concentration but triggered by strain (Plate B). The colours indicate the morphology of the observed sprouts for each set of parameters. In both cases the black dots correspond to situations where vessel grows very slowly, not being able to reach a few cell widths in length after 14.5 hours. The red dots correspond to deformed vessels (triangular or with variable thickness), the orange dots correspond to sprouts that are straight but with a significantly thickened parental vessel, and the green dots correspond to well formed sprouts without appreciable thickening of the parental vessel. We observe that the proliferation is able to extend the short sprouts.

In all these types of regulation, we observe that endothelial cell proliferation is able to prevent the sprouts from breaking when the traction force and adhesion are high. In the same way, endothelial cell proliferation is able to cause the sprout to grow indefinitely at lower adhesion and/or traction, when initially the sprout achieved a maximum length and did not break up (Figs 2C and 3C). In Fig 8 we plot the equivalent graphics of Figs 6A and 7A but for adhesion *α* = 0.31 KPa and maximum traction equal to 3.0 KPa. For these parameters, in the absence of proliferation the sprout would not break and only reach 40 *μ*m (Fig 2) after 14.5 hours. For proliferation regulated by VEGF concentration, we observe that for low proliferation the sprouts still grow very slowly. We mark in black in Fig 8A the situations where the vessel is not able to extend 60 *μ*m (approximately six cell lengths) during our simulation time. We never observe breaking as in Figs 6A and 7A. As the proliferation rate of the endothelial cell increases, we observe first well formed long vessels (in green), then thick parental vessels (in orange), and finally malformed triangular vessels (in red), similarly to what is observed in Fig 6. For proliferation regulated by VEGF concentration but triggered by strain the thickening of the parental vessel is not present (Fig 8B). We clearly observe in Figs 4, 5, 6, 7 and 8 that the largest region in parameter space with well formed sprouts is obtained always when the proliferation is triggered by endothelial cell strain and its rate grows with VEGF concentration.

The endothelial cell strain ∇^2^ *w* = −*αϕ* + *χ* ^*t*^ is composed of two terms: the first term is regulated by the adhesion *α* and becomes positive when the order parameter is below *ϕ* = 1, and the second term depends on *χ* ^*t*^, being related to the pulling force exerted upon the stalk cells when the tip cell contracts. Therefore, if the proliferation is triggered by strain, there are two ways to prompt cell replication: either the contraction of the tip cell stretches the tissue, or there is a lower density of cells (*ϕ* < 1) at some point. The latter scenario is clearly observed in endothelial cell cultures where contact inhibition is present, since in these cultures cell proliferation only occurs if there is space between the cells.

We conclude that in our sprouts the tip cell has the role of creating a tension in the cells that follow its lead. On those first stalk cells, this tension produces strain and/or empty spaces, inevitably triggering cell proliferation. The new cells occupy the space behind the tip, the tension decreases, and the process restarts.

In this work we introduced a new phase-field model for angiogenesis that is able to describe sprout development as a function of both chemical factors (VEGF levels) and the mechanical environment of the surrounding tissue. The presented model is very compact, with the dynamics of the vessel being dependent on only 6 parameters (*α*, *L*_0_, *μ*_1_, *ϵ*, *ρ*_*ϕ*_ and *M*) plus the location and profile of the tip cells’ traction fields and the definition of the endothelial cell proliferation, both of which can be obtained experimentally.

The 6 parameters can be directly related to experimental measurements. The values of *L*_0_ and *μ*_1_ depend directly from the Young’s modulus and shear ratio of the vessel and ECM (see fourth section of S1 Text) and the coefficient *α* can be obtained from the measurement of the adhesion forces per unit area between two endothelial cells (either by atomic force microscopy [80] or by traction force microscopy [81]). The mobility coefficient of the simulation can be chosen by matching the velocity of the endothelial cells in the simulation to the velocity observed experimentally (as it is done in the present article), while the value of the *ρ*_*ϕ*_ defines the maximum length of a single sprout if there is no proliferation. The results of the model are independent of the value chosen for *ϵ*, which is the width of the capillary wall in the simulation (in phase-field models the interface width is the smallest length scale in the simulation [65]). A different *ϵ* can be compensated by altering the value of *ρ*_*ϕ*_ in order to match the observed vessel length. This model consists of the integration of a single partial differential equation where one of the parameters, *w*, related to the strain of the microenvironment, is obtained at every iteration as a function of the applied forces. The absence of several rule based mechanisms in the model will permit in a short time its implementation within several PDE solving engines, allowing it to run in parallel computing environments. This will allow running larger systems, and even consider its extension to 3D, which will enable the study of lumen formation and aneurisms. Also, this framework will allow the study of the action of flow in vessel remodelling, since we can couple local forces with cell movement to obtain the final vessel morphology. Moreover, the use of phase-field modelling permits the inclusion of more stable minima by alteration of the Ginzburg-Landau potential (in the first section of S1 Text). The new minima can describe the presence of other tissues, or of more complex ECM formations such as a basement membrane, for example. The formation of a basement membrane occurs around mature vessels, and ends up by surrounding them. Phase-field has been used to model surfactant phases that occupy the boundary between two other phases [82], and also surfactant phases that are chemically formed during the evolution of the simulation [83], similarly to what occurs with the basement membrane. Therefore the present modelling strategy could in principle be extended to model a basement membrane as a third component with specific mechanical properties. Such a model could be used to study how the basement membrane creation/degradation dynamics impacts vessel extension and regression.

The ECM is a very complex system *per se*, which can be described as a mixture of two phases: the interstitial fluid and the ECM fibres [84]. At the scale of the problem discussed in this article, the ECM can be viewed morphologically as a single phase. However, since the diffusible growth factors diffuse on the interstitial fluid, we could expect that the characterisation of the diffusion of VEGF should take into consideration a more complex description of the ECM. This is certainly a very relevant direction of future research. Also, the capture of the heavier VEGF isoforms by the matrix fibres [25, 71] could be included in the model (similarly to what was done in [25]) in a way to improve the characterisation of the VEGF concentration in live tissues. Descriptions of ECM as a complex two-phase system have been carried out in tumor growth models using mixture theory [85], and can also be implemented into a phase-field description.

The present model can also be used to study the role of tissue mechanics during vessel sprouting, extension and anastomosis in large vessel networks. We are currently working on this topic where we will compare quantitatively the morphology of the resulting networks with sprout formations observed *in vitro*. When several vessel sprouts are present, the Notch-Dll4 signalling pathway that regulates the activation of endothelial cells has to be considered [52, 60]. This can be done by forbidding the activation of a new tip cell (i.e. the inclusion of a traction force field as described in this article) at any point that is closer than two cell diameters from any center of an already existing traction force field. The implementation of this exact mechanism was already considered in [25].

In the present work we used this method to couple the stress exerted by the tip cells to the final morphology observed in order to highlight the importance of the regulation of cell proliferation by stress in sprouting angiogenesis. We demonstrated that well formed sprouts are possible for the largest region of parameter space if proliferation is controlled by VEGF concentration specifically in sites where cells are under strain. The sites of high positive strain identify the localisations where new endothelial tissue is required for the sprout to grow effectively.

The triggering of endothelial cell proliferation by the strain is clearly supported in the literature [18, 76–78], and suggests a mechanism for sprout extension, where the role of the tip cell is to create the strain that sets off cell proliferation close to the front of the growing vessel.

## Supporting Information

S1 Text Supporting documentation: In this text we present the derivation of the model’s equations, justify the parameters used and describe the experimental methods. (PDF)
