# Correction: The Force at the Tip - Modelling Tension and Proliferation in Sprouting Angiogenesis

**DOI:** 10.1371/journal.pcbi.1004616

**Published:** 2015-11-30

**Authors:** The PLOS Computational Biology Staff

The following sentence should be included in the funding statement of this article:

"HG and RS thank the support of Fundação para a Ciência e Tecnologia through the project with reference number UID/NEU/04539/2013."
