# Supplementary material for: The Force at the Tip - Modelling Tension and Proliferation in Sprouting Angiogenesis
Source: PLoS Comput Biol. 2015 Aug 6;11(8):e1004436. doi: 10.1371/journal.pcbi.1004436 (PMC4527825; doi:10.1371/journal.pcbi.1004436)
Supplement: S1 Text — (PDF) [file pcbi.1004436.s001.pdf]

Supporting Information

1. Free energy

In the present model, the configuration of the vascularised tissue is represented by an order parameter $\phi(\mathbf{r})$ that distinguishes the two phases of the system, being equal to 1 inside the capillary and -1 outside [1]. To describe the elastic behaviour we include a displacement field $\mathbf{u}(\mathbf{r})$ that represents the local displacement with respect to the reference unstressed configuration. Akin to what happens in phase separation of elastic blends [2, 3], the order parameter relaxes much slower than the mechanical environment, and therefore, we consider that all the forces are constant in time when describing the evolution of ϕ. Our strategy starts by defining a free-energy functional of the order parameter and the displacement field. For this specific free energy we will start by obtaining the displacement as a function of the order parameter. We can then calculate the functional derivative of the free energy with respect to the order parameter, i.e. the chemical potential of the system. The evolution of the order parameter depends of this chemical potential in the following manner [1, 4]:

$$\frac{\partial \phi}{\partial t} = M\nabla^2 \frac{\delta F}{\delta \phi} + \text{prolif.} \; ,$$

where M is the mobility coefficient and the last term corresponds to the proliferation of the tissue cells.

The free energy of the system is then the sum of F_ϕ, the energy associated to the system configuration, with the elastic free energy associated to the displacements, F_{el}, and with the free energy related to the forces exerted by the cells in the system $F_{\mathfrak{f}}$. Therefore:

$$F[\phi(\mathbf{r}),\mathbf{u}(\mathbf{r})] = F_\phi[\phi(\mathbf{r})] + F_{el}[\mathbf{u}(\mathbf{r})] + F_{\mathfrak{f}}[\phi(\mathbf{r}),\mathbf{u}(\mathbf{r})] = \int d\mathbf{r} \left[\rho_\phi \left(-a\frac{\phi^2}{2} + \frac{\phi^4}{4} + \frac{\epsilon^2}{2}\left(\nabla\phi\right)^2 \right) + \frac{1}{2}\boldsymbol{\sigma} : \boldsymbol{\varepsilon} - \chi \nabla \cdot \mathbf{u} \right] \; . \quad (1)$$

In this equation $\rho_\phi \left(-\frac{a\phi}{2} + \frac{\phi^4}{4} + \frac{\epsilon^2}{2}\left(\nabla\phi\right)^2 \right)$ is the contribution to the free energy density that is associated to the order parameter. This term is responsible for the interface between the two tissues [1], and leads to a Cahn-Hilliard term [4] in the final equation. In here, ρ_ϕ is proportional to the free energy density of a pure phase, a is a constant and ϵ is the width of the interface, which in phase-field models is also associated to the surface tension. This term guarantees the existence of two minima in the free energy (corresponding to the two phases) and includes an energy penalty for spatial variations of the order parameter, i.e. a surface tension.

The elastic contribution for the feee-energy density is given by $\frac{1}{2}\boldsymbol{\sigma} : \boldsymbol{\varepsilon}$, where the strain tensor elements are

$$\varepsilon_{ij} = \frac{1}{2}\left(\partial_i u_j + \partial_j u_i\right) \; .$$

We consider the tissues to be homogeneous and isotropic for simplicity, though a similar derivation of the model can be also obtained for other constitutive relations. For isotropic and homogeneous materials the stress tensor elements are given by

$$\sigma_{ij} = K\delta_{ij}\varepsilon_{kk} + 2\mu\left(\varepsilon_{ij} - \frac{1}{d}\delta_{ij}\varepsilon_{kk}\right) \; .$$

In this equation K is the compressibility modulus, μ the rigidity modulus, d the dimensionality of the system, and δ_{ij} the Kronecker symbol.

The free energy density associated to the force exerted by the cells, $-\chi \nabla \cdot \mathbf{u}$, will be discussed below.

Since the displacement field relaxes much faster than the order parameter, we can consider a situation of mechanical equilibrium, i.e. $\frac{\delta F}{\delta u_i} = 0$. Calculating the functional derivative of F_{el} with respect to the displacement we obtain

$$\frac{\delta F_{el}}{\delta u_i} = -\partial_j \left[K \delta_{ij} \varepsilon_{kk} + 2\mu \left(\varepsilon_{ij} - \frac{1}{d} \delta_{ij} \varepsilon_{kk} \right) \right] = -\partial_j \sigma_{ij} \ .$$

Therefore, since the mechanical equilibrium law states that $\frac{\delta F}{\delta u_i} = \partial_j \sigma_{ij} + \mathfrak{f}_i = 0$, the force density exerted by the cells has to obey $-\mathfrak{f}_i = \frac{\delta F_{\mathfrak{f}}}{\delta u_i} = \partial_i \chi$. And so, the gradient of the field $\chi(\mathbf{r})$ if proportional to the force density.

There are two important forces in the system: the traction exerted by the tip cell, $\mathfrak{f}_i^t = -\partial_i \chi^t$, and the force exerted when one cell adheres to its neighbour. This force of adhesion mimics the attraction between neighbouring cells that is conveyed by the adhesion molecules [5], and can be written as $\mathfrak{f}_i^a = -\partial_i \chi^a = \alpha \partial_i \phi$, where α is a constant. This force points in the direction of increasing ϕ and has the role of balancing the pulling exerted at the tip during angiogenesis [6]. The corresponding field $\chi^a(\mathbf{r})$ is then given by $\chi^a(\mathbf{r}) = -\alpha \phi(\mathbf{r})$. Notice that while the adhesion $\chi^a(\mathbf{r})$ is a function of the order parameter, we assume that the traction force exerted by the tip cell is not a function of ϕ. At the tip, the cell will contract and keep the traction force for some time while the tissue adapts to that force. During that time, $\chi^t(\mathbf{r})$ is a constant field.

2. Evolution of the order parameter

Considering a small difference between the rigidity moduli of the two phases, such that $\mu = \mu_0 - \mu_1 \phi$, with $\mu_1 \ll \mu_0$, we will expand the derivative of the free energy with respect to the displacement in powers of μ_1. In the same way, the displacement field $\mathbf{u}(\mathbf{r})$ and the free energy function can also be split into components of order zero and components of order one: $\mathbf{u}(\mathbf{r}) = \mathbf{u^0}(\mathbf{r}) + \mathbf{u^1}(\mathbf{r})$ and $F[\phi(\mathbf{r}), w(\mathbf{r})] = F^0[\phi(\mathbf{r}), w(\mathbf{r})] + F^1[\phi(\mathbf{r}), w(\mathbf{r})]$.

Collecting the terms of first order, we obtain:

$$\frac{\delta F_{el}^0}{\delta u_i} = -\left(K - \frac{2\mu_0}{d} \right) \partial_{ij} u_j^0 - \mu_0 \left(\partial_{ij} u_j^0 + \partial_{jj} u_i^0 \right) = -\left(K - \frac{2\mu_0}{d} + 2\mu_0 \right) \partial_{ijj} w = -L_0 \partial_{ijj} w \ ,$$

where $L_0 = \left(K - \frac{2\mu_0}{d} + 2\mu_0 \right)$ is a constant and we define a field w such that $\mathbf{u^0}(\mathbf{r}) = \nabla w$.

The derivative of the whole free energy yields $\frac{\delta F}{\delta u_i} = 0$, giving $-L_0 \partial_{ijj} w + \partial_i \chi = 0$. This equation is satisfied if

$$L_0 \nabla^2 w = \chi = -\alpha \phi + \chi^t \ . \tag{2}$$

Using equation (2), and the definition of w, we can re-write $F_{el}^0[\phi(\mathbf{r}), w(\mathbf{r})] + F_{\mathfrak{f}}^0[\phi(\mathbf{r}), w(\mathbf{r})]$ in equation (1) to get:

$$F_{el}^0[\phi(\mathbf{r}), w(\mathbf{r})] + F_{\mathfrak{f}}^0[\phi(\mathbf{r}), w(\mathbf{r})] = -\int d\mathbf{r} \frac{1}{2} \chi \nabla^2 w = -\int d\mathbf{r} \frac{\chi^2}{2L_0} \ .$$

Repeating the process for the first order term in the free energy we observe that all the terms in this order that are proportional to $\mathbf{u^1}(\mathbf{r})$ cancel out, giving simply

$$F_{el}^1[\phi(\mathbf{r}), w(\mathbf{r})] + F_{\mathfrak{f}}^1[\phi(\mathbf{r}), w(\mathbf{r})] = -\int d\mathbf{r} \mu_1 \phi \left(\partial_{ij} w \ \partial_{ij} w - \frac{1}{d} (\nabla^2 w)^2 \right) \ .$$

Putting all together, we can finally calculate the partial derivative of the free energy with respect to the order parameter:

$$\frac{\delta F}{\delta\phi} = \rho_\phi\left(-\phi+\phi^3-\epsilon^2\nabla^2\phi\right) + \frac{\alpha\chi^t}{L_0} - \mu_1\left(\partial_{ij}w\;\partial_{ij}w - \frac{1}{d}(\nabla^2 w)^2\right) + \frac{2\mu_1\alpha}{L_0}\left([\nabla]^{-2}\partial_{ij}(\phi\;\partial_{ij}w) - \frac{\phi}{d}\nabla^2 w\right)\;,$$

where $[\nabla]^{-2}$ is the inverse laplacian operator, and the constant a can be set for convenience such that $a - \frac{\alpha^2}{L_0\rho_\phi} = 1$, such that the two minima of the order parameter fall at $\phi = 1$ and $\phi = -1$.

Finally we can obtain the equation for the evolution of ϕ by $\frac{\partial\phi}{\partial t} = M\nabla^2\frac{\delta F}{\delta\phi}$ (to which we will add the proliferation term below), yielding

$$\frac{\partial\phi}{\partial t} = M\left\{\nabla^2\left[\rho_\phi\left(-\phi+\phi^3-\epsilon^2\nabla^2\phi\right) + \frac{\alpha\chi^t}{L_0}\right] - \mu_1\nabla^2\left(\partial_{ij}w\;\partial_{ij}w - \frac{1}{d}(\nabla^2 w)^2\right) + \frac{2\mu_1\alpha}{L_0}\;\partial_{ij}\left[\phi\;\left(\partial_{ij}w - \frac{\delta_{ij}}{d}\nabla^2 w\right)\right]\right\}\;. \tag{3}$$

3. Numerical Implementation

In a square domain of 120×120 μm, with a lattice unit of 1 μm, we start with a single vessel of width 10 μm, where $\phi = 1$ inside that vessel and $\phi = -1$ outside. The VEGF initial concentration $V(\mathbf{r}, 0)$ is set to have a constant gradient, with $V = 1$ at the system's right boundary, and $V = 0$ at its left boundary.

The VEGF concentration in the system evolves according to the following diffusion equation:

$$\frac{\partial V}{\partial t} = D_V\nabla^2 V - \alpha_V V\phi\Theta(\phi)\;,$$

where D_V is the diffusion constant of VEGF, α_V is the VEGF consumption rate by the endothelial cells, and $\Theta(\phi)$ is the Heaviside function. The penetration width of VEGF in the endothelial tissue is given by $\sqrt{D_V/\alpha_V}$ [1]. This equation is solved by finite differences and with fixed values of V at the left and right boundary conditions.

We start by running the simulation for a sufficient number of time steps until the VEGF field becomes stationary. Then, at the vessel's mid-height, on its right surface, we will include the traction force field originated by the tip cell action.

The evolution of the order parameter, according to equation (3), depends on the zeroth order displacements through the field w. This field is a function of ϕ and of the tip cell traction (according to equation(2)), and so we start by obtaining $\chi^t(\mathbf{r})$ numerically. The tip cell exerts a compressible force [7, 8], and, hence, we consider a putative compressible traction force with the direction of the gradient of VEGF applied at the vessel tip, $\mathbf{f_1^t}$. We set the magnitude of this component to decrease exponentially (as a gaussian function) far away from the application point. In the next figure we plot the magnitude of $\mathbf{f_1^t}$ as a function of the position.

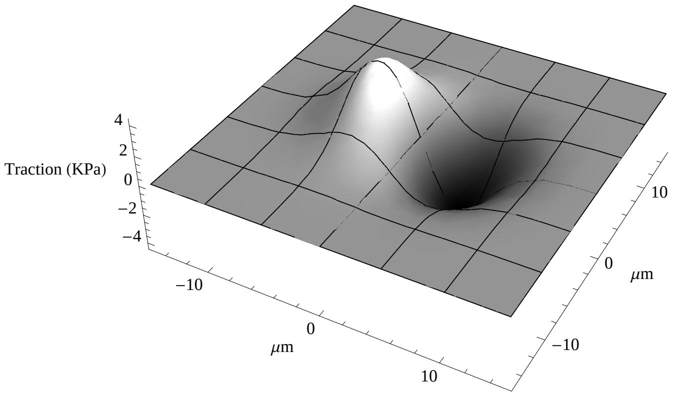

In the way that the model is designed, the traction is proportional to the gradient of a localised field χ^t. That is not the case for the putative traction $\mathbf{f_1^t}$, which only has a component in the direction of the VEGF gradient, implying that the field χ^t would be constant and different from zero (and therefore, not localised) along the direction perpendicular to the gradient of VEGF. We correct the traction field $\mathbf{f_1^t}$ by adding a small non-constant component in the direction perpendicular to the gradient of VEGF. This small component is calculated indirectly by first obtaining a field $\chi^t(\mathbf{r})$ from $\mathbf{f_1^t}$ by solving the following equation:

$$\nabla^2\chi^t = -\nabla \cdot \mathbf{f_1^t} \ . \tag{4}$$

This equation is solved using finite differences with the boundary condition of $\chi^t = 0$ at the boundaries. In this way, the χ^t obtained is localised, and the traction given by $\mathbf{f^t} = -\nabla\chi^t$ that we are in fact simulating, is also localised, compressible, and has a small component perpendicular to its main direction of action (see Figure 2A, main article). For other more complex force distributions (e.g. the traction fields measured experimentally [7, 8]), the $\chi^t(\mathbf{r})$ can be also obtained using equation (4), by replacing $\mathbf{f_1^t}$ for the traction field of interest.

The field $\chi^t(\mathbf{r})$ is kept constant for a certain number of time steps t_{cell} while the order parameter evolves. After that time, the capillary wall has moved and the field $\chi^t(\mathbf{r})$ is again calculated using equation (4), but centred at the new position of the capillary wall.

To simulate the evolution of the order parameter, we solve equation (3) with a term describing proliferation. In this equation w is obtained by solving equation (2) numerically at every time step. We use periodic boundary conditions and set the spatial average of w to be equal to zero (notice that the value of the average of w does not affect the displacement field).

As explained in the main text, the proliferation $\alpha_p[V, \phi, w]$ at a point is the average of the proliferation in an area the size of a cell, being given by

$$\alpha_p[V, \phi, w] = \frac{\int_\Omega p(V, w)\Theta(\phi) d\mathbf{r}}{\int_\Omega \Theta(\phi) d\mathbf{r}} \ ,$$

where Ω is the area of a cell (we consider a circle of radius 5 μm), $\Theta(\phi)$ is the Heaviside function, and $p(V, w)$ is the proliferation function used. In the main article, we consider that the proliferation may depend either on V or on the strain, i.e. on $\nabla \cdot \mathbf{u^0} + \alpha = \nabla^2 w + \alpha = \alpha(1-\phi) + \chi^t$. For the first case we characterise the proliferation,

i.e. $p(V, w) = p(V)$, by two constants: the limit VEGF concentration, L_V and the maximum proliferation, M_P. Therefore, $p(V)$ is given by

$$p(V) = \begin{cases} \frac{2M_P V}{L_V} & , \quad V < L_V \\ 2M_P & , \quad V \geq L_V \end{cases} . \tag{5}$$

In the definition of $p(V)$, the maximum cell proliferation M_P is multiplied by a factor 2 because the order parameter has to be increased by two units (from $\phi = -1$ to $\phi = 1$) for a new capillary region to form. Equation (5) is the dependence for the proliferation used in Figure 6 of the main text. For Figure 7, the proliferation of equation (5) is multiplied by the Heaviside function $\Theta(\nabla^2 w(\mathbf{r}) + \alpha - S_m)$, where S_m is the minimum strain that is required to exist proliferation.

When the proliferation depends on $\nabla^2 w$ (Figure 4) the proliferation function used is similar to equation (5) but with the VEGF concentration replaced by the strain, and the L_V replaced by the limit strain L_S. For Figure 6 of the main text we require that the proliferation is only different from zero if there is a minimum concentration of VEGF V_m present, i.e. $p(w)$ is multiplied by $\Theta(V(\mathbf{r}) - V_m)$.

In Figures 5 and 7 the values used for V_m and S_m follow the reasoning that a small VEGF concentration (in Figure 5) or a small strain (in Figure 7) is sufficient to trigger endothelial cell proliferation. In this way, the values used for V_m and S_m are the minimum values tested in Figures 6 and 4 respectively, i.e. $V_m = 0.05$ and $S_m = 0.05$.

Having w and $\alpha_p[V, \phi, w]$, equation (3) is finally integrated using a finite different scheme with periodic boundary conditions.

4. Parameters

The goal of the present article is to introduce a compact and simple to implement phase-field model for sprouting angiogenesis able to describe sprout morphology as a function of the mechanical characteristics of the tissue and cells. The model is also used to provide insight to the possible factors regulating endothelial cell proliferation in the sprouts. In the present article we are not attempting to model specific experiments, and therefore we will use rough estimates for the different parameters of the model.

We first present the table of parameters used (denoting the extra-cellular matrix by ECM and the endothelial cells by EC), and below we discuss the choices made when setting these parameter values.

Parameter	Value	Reference/Comment
ECM Young's Modulus	3.0×10^3 Pa	see [9]
ECM Poisson Ration	0.13	see [9]
EC Young's Modulus	4.0×10^2 Pa	see [10]
EC Poisson Ration	0.49	see [8]
μ_0	7.3×10^2 Pa	Average rigidity moduli
μ_1	6.0×10^2 Pa	Difference between rigidity moduli of EC and ECM
L_0	4.74×10^3 Pa	Given by $K + 2\mu_0 - \frac{2\mu_0}{d}$, where K is the average compressibility
ϵ	1 μm	Order of magnitude of the capillary wall width [1]
ρ_ϕ	4.74×10^3 J/m^3	Free energy density of phase-separation term, equal to L_0
M	8.1×10^{-18} m/J.s	Mobility obtained by setting the timescale of the problem
t_{cell}	4.3 min	Time between two calculations of the tip traction field
D_V	4.2×10^{-13} m^2/s	Diffusion constant of VEGF, fastest process in the simulation [1, 11]
α_V	3.9×10^{-3} s^{-1}	So that $\sqrt{D_V/\alpha_V}$ is on the order of the cell diameter [1]

The constants reflecting the mechanical characteristics of the tissues, i.e. the Young's and rigidity moduli, are used to calculate the parameters μ_0 (average of both rigidity moduli), μ_1 (difference between the rigidity moduli), K (average compressibility modulus) and L_0 (given by $L_0 = K + 2\mu_0 - \frac{2\mu_0}{d}$, where $d = 2$ is the dimensionality of the system). In the particular choice of parameters used in this example, $\mu_1 \lesssim \mu_0 \ll L_0$. Though the terms of higher order that were disregarded in the derivation of equation (3) could in principle still be large enough to alter some of the the quantitative results presented, they are not expected to alter the very clear qualitative result of how the proliferation should be regulated for the generation of well-formed sprouts.

The role of the first term in the free energy, $F_\phi[\phi(\mathbf{r})]$, is the formation of the interface between the capillary and the extracellular matrix. Small variations in the profile of the order parameter across the interface with respect to equilibrium are rapidly corrected by this term. The parameter ρ_ϕ determines how fast the equilibrium shape of the interface is achieved. However, if ρ_ϕ is very large, the dynamics of the system is governed by the Cahn-Hilliard part of equation (3) and not by the elastic component or the endothelial cell traction. Therefore, ρ_ϕ should be large enough for the interface to be formed rapidly, but small enough for sprout extension being determined by the mechanical properties of the tissues and not by the capillary wall surface tension. This balance occurs when the order of magnitude of $F_\phi[\phi(\mathbf{r})]$ is the same as the order of magnitude of $F_{el}[\mathbf{u}(\mathbf{r})]$ [2, 3], and therefore, we set $\rho_\phi = L_0$.

The value of the mobility M is chosen to match approximately the timescale of sprout growth *ex vivo*. In Figure 3 of the main text we observe that individual cells are able to migrate alone in the matrix. The maximum distance these cells are able to cover in 24 hours is approximately independent of the initial amount of VEGF in the matrix (provided it is not too large, so that it saturates the cell receptors). All these cells travel at approximately 9.5 μm/hr. We simulated a single sprouting event, with no proliferation, setting the adhesion to $\alpha = 4.7 \times 10^2$ Pa and maximum traction force 3.0×10^3 Pa. With these parameters, and according to Figure 2C from the main document, the tip cell separates from the main vessel and migrates in the direction of increasing VEGF levels. We observed the cells

migrate at constant velocity and we altered the time scale of our simulation (given by the mobility M) so that the cell migration velocity matches the value observed experimentally.

Finally, the VEGF diffusion occurs in a faster timescale than endothelial cell rearrangement and migration. The large value for the VEGF diffusion constant [1, 11] guarantees that the VEGF profile achieves the equilibrium for a particular sprout morphology. The value of VEGF consumption α_V is chosen so that the VEGF penetration width $\sqrt{D_V/\alpha_V}$ is on the order of the cell diameter [1].

5. *Experimental Model: Matriplug Assay*

Male Wistar rats (6 months old) were obtained from local breeding colonies maintained at the animal facility of the Faculty of Medicine - University of Coimbra. The animals were subjected to a constant daily cycle of 12 hours of light and 12 hours dark with constant temperature (22 - 24°C) and humidity (40 - 70%). Rats were given free access to water and to standard commercial pellet chow diet (AO4; Panlab, Barcelona, Spain).

To model VEGF-driven *in vivo* angiogenesis, a subcutaneous matrigel plug assay was performed, as previously described [12], with modifications. Briefly, growth factor reduced Matrigel (BD Biosciences, Bedford, MA, USA) was thawed at 4°C overnight to become liquid. After anaesthetised, each rat was injected subcutaneously in the back, 1 cm off the midline, with 0.6 mL of liquid Matrigel supplemented with heparin (Sigma, St Louis, MO, USA), with or without VEGF (R&D Systems, Minneapolis, MN, USA) in the indicated concentrations.

Seven days after implantation animals were anaesthetised and injected through the jugular vein with an Evans Blue (Sigma-Aldrich, St.Louis, MO, USA) - PBS solution (100mg/Kg of body weight). Evans blue is a dye that combines with plasma albumin, allowing for detection of functional vessels. Thirty minutes later, the animals were killed by cervical dislocation and the matrigel plugs were enucleated.

Removed matrigel plugs were fixed in with 4% paraformaldehyde for 10 minutes, washed, embedded in OCT (Tissue-Tek, Histolab, Sweden) and stored frozen at -80°C until further analysis. Vascular density was determined by immunofluorescence on 5 μm cryosections. The samples were permeabilized with 1% Triton X-100 (v/v) for 10 minutes and blocked with 5% Bovine Serum Albumin (BSA) for 1 hour prior the incubation with a marker for endothelial cells, primary antibody rabbit Anti-von Willebrand Factor (vWF) (Dako, Glostrup, Denmark) overnight at 4°C. The samples were then rinsed three times with PBS and incubated with 1:5.000 DAPI (Invitrogen, Carlsbad, CA, USA) and 1:100 Alexa Fluor® 488-conjugated goat anti-rabbit (Invitrogen, Carlsbad, CA, USA) for 1 hour at room temperature. The samples were then washed with PBS and mounted with Mowiol® 4-88 Reagent (Calbiochem, San Diego, CA, USA). The images were collected by confocal microscopy using a Zeiss LSM 710 Confocal microscope (Carl Zeiss MicroImaging GmbH, Jena, Germany).

6. *Experimental Model: Aortic Ring Assay*

Reagents. Unless otherwise stated, all reagents were purchased from Merck Darmstad (Germany), Sigma-Aldrich (EUA) or Pancreac Qumica SA (Spain).

Animal models. In this work were used 8-12 weeks old Wistar rats, from our breeding colonies at the Faculty of Medicine, University of Coimbra. The animals were kept (2 per cage) under standard ventilation, temperature (22-24°C), humidity (50-60%) and light (12 hours light/ 12 hours darkness) with free access to water and food (standard diet AO4, Panlab, Barcelona). The experimental protocol was approved by the local Institutional Animal Care and Use Committee and all the procedures were performed by licensed users (FELASA).

Ex vivo study of angiogenesis: growth of sprout-like structures. Animals were sacrificed by cervical dislocation and the aorta was harvested and washed twice in Opti-MEM (31985-047, Gibco, United kingdom), without serum and supplemented with 1X Penicillin-Streptomycin (10378-016, Gibco, United kingdom). The aorta was cut in rings of $\sim$0,5 mm using a scalpel blade and the rings were incubated in a petri dish (35 mm) with Opti-MEM without serum and supplemented with 1X Penicillin-Streptomycin, overnight at 37°C 5% CO2. The aortic rings were embedded in a collagen matrix (08-115, Millipore, USA) in different plates: 96 wells (counting experiences); 4 and 8 wells (immunohistochemistry). After polymerization, Opti-MEM growth medium supplemented with 2.5% FBS (10082-147, Gibco, United kingdom), 1X Penicillin-Streptomycin and recombinant rhVEGF (293-VE, R&D Systems, USA) was added (200 and 500 μL, respectively) and half of the volume was replaced every second day until the end of the experience.

Ex vivo study of angiogenesis: live confocal imaging. Live imaging of growing sprouts was performed using zeiss confocal microscopy (633 nm) with 10x of magnification and at stable temperature. The acquisitions were made at different time points of the experience and always in the same position to allow sprout follow.

Statistical analysis. Results are presented as mean ±SEM. One way ANOVA-test was used to determine statistical differences. Post-hoc Tukey test was performed to assess the differences between groups. $P < 0.05$ was considered significant.

7. *Examples of Single Runs*

In this section we include the some graphics of single runs for different parameters.

First we analyse the growth of a single sprout for different values of the proliferation rate. In our model there is a balance between the force of the tip cell and the surface tension term (the first term of equation (1) of the main text). If there is no proliferation, the growth becomes sub-linear and it is effectively arrested. In the next picture we plot the position of the tip cell as a function of time for the case where there is arrest of vessel growth ($\alpha = 0.37$ KPa and traction 2.7 KPa). We present four lines that correspond to different maximum proliferation rates (we use the case of proliferation proportional to VEGF concentration and triggered by strain).

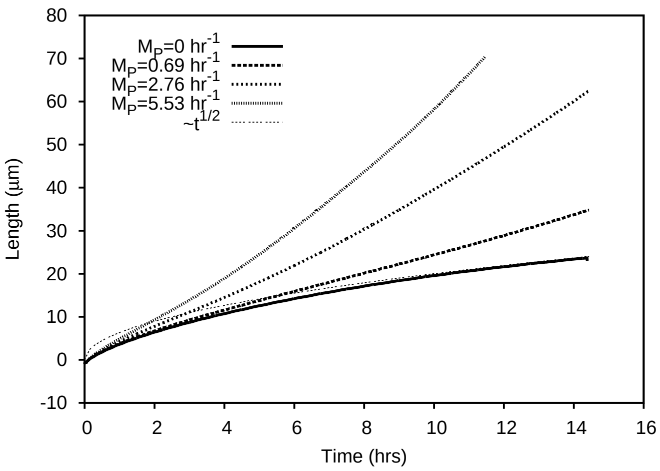

In this figure we observe that when there is no proliferation the vessel grows very slowly (proportional to $\sim t^{1/2}$). After 12 hours the tip cell is already moving at a very slow speed: only 10 μm in 8 hours, and it is still slowing down. Though the vessel does not effectively stop, the velocity decreases so much that in an experiment the cell would end up moving less than its length per day. Compare with what occurs when there is proliferation, where the vessel is able to maintain a constant growing velocity. The proliferation is always able to increase the velocity of the growing sprout.

Then we observe the sprout length for different values of ρ_ϕ. As we increase ρ_ϕ the surface tension increases and the vessel's final length will be shorter. Lower values of ρ_ϕ would make easier for the tip cell to leave the growing sprout.

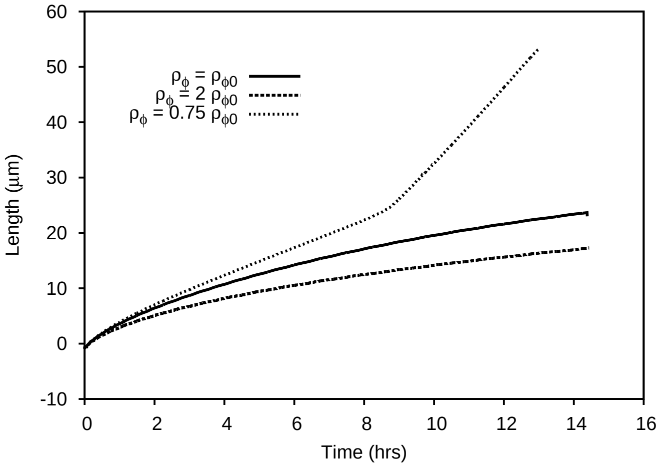

In this figure we show the position of the tip cell for a sprout with no proliferation as a function of time for 3 values of ρ_ϕ: equal to the value used in the paper, two times larger (resulting in a shorter vessel) or three quarters of the initial value (the tip cell starts moving faster and eventually breaks apart after 8 hours). The value of the adhesion and the traction is the same as in the previous figure.

Finally we show the length of the sprout for different values of the ECM's Young's modulus. The ability of endothelial cells to migrate and orient depends on the value of the Young's modulus in a non-monotonous manner [6] (i.e. there is an optimal value for the Young's modulus for network formation). In the figure below we present the

growth in time of the position of the front of a growing sprout with no proliferation for four different values of the Young's modulus of the ECM: $E = 0.2E_0$, $E = 0.6E_0$, $E = E_0$ and $E = 1.5E_0$, with $E_0 = 3.0$ KPa, the value used in the article. The values for the adhesion and traction are the same used in the previous two figures.

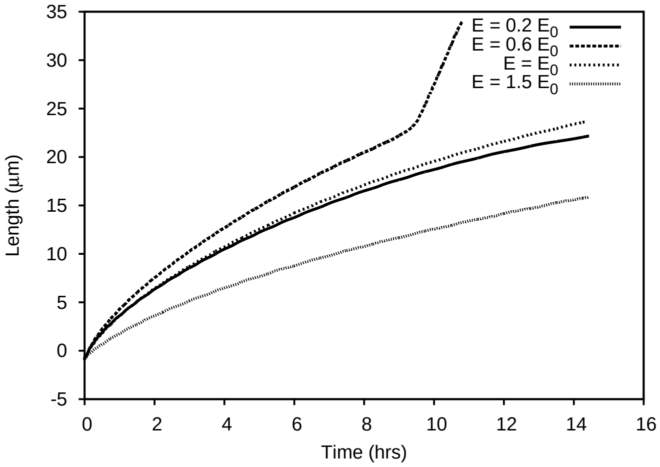

In this figure we observe the non-monotonous behaviour expected: the sprout achieves the faster growth at the intermediate Young's modulus $E = 0.6E_0$ (the tip cell even splits from the main vessel after approximately 9 hours). High values and low values of the Young's modulus lead to shorter vessels, as expected.

---

[1] Travasso, R. D., Poiré, E. C., Castro, M., Rodríguez-Manzaneque, J. C., Hernández-Machado, A. (2011). Tumor angiogenesis and vascular patterning: a mathematical model. PloS one, 6(5), e19989.

[2] Nishimori, H., Onuki, A. (1990). Pattern formation in phase-separating alloys with cubic symmetry. Physical Review B, 42(1), 980.

[3] Onuki, A. (2002). Phase transition dynamics. Cambridge University Press.

[4] Bray, A. J. (2002). Theory of phase-ordering kinetics. Advances in Physics, 51(2), 481-587.

[5] M. Nonomura (2012) Study on multicellular systems using a phase field model. PloS one 7, e33501.

[6] Merks, R. M., Perryn, E. D., Shirinifard, A., Glazier, J. A. (2008). Contact-inhibited chemotaxis in de novo and sprouting blood-vessel growth. PLoS computational biology, 4(9), e1000163.

[7] Reinhart-King, C. A., Dembo, M., Hammer, D. A. (2008). Cell-cell mechanical communication through compliant substrates. Biophysical journal, 95(12), 6044-6051.

[8] Califano, J. P., Reinhart-King, C. A. (2009). The effects of substrate elasticity on endothelial cell network formation and traction force generation. In Engineering in Medicine and Biology Society, 2009. EMBC 2009. Annual International Conference of the IEEE (pp. 3343-3345). IEEE.

[9] Raub, C. B., Putnam, A. J., Tromberg, B. J., George, S. C. (2010). Predicting bulk mechanical properties of cellularized collagen gels using multiphoton microscopy. Acta biomaterialia, 6(12), 4657-4665.

[10] Feneberg, W., Aepfelbacher, M., Sackmann, E. (2004). Microviscoelasticity of the apical cell surface of human umbilical vein endothelial cells (HUVEC) within confluent monolayers. Biophysical journal, 87(2), 1338-1350.

[11] Schugart RC, Friedman A, Zhao R, Sen CK (2008) Wound angiogenesis as a function of tissue oxygen tension: A mathematical model. Proc. Natl. Am. Sci. USA 105:2628-2633.

[12] Malinda, K. M. (2009). In vivo matrigel migration and angiogenesis assay. In Angiogenesis Protocols (pp. 287-294). Humana Press.
